# Supplementary material for: Polyketide synthase gene clusters in the ericoid mycorrhizal fungus Oidiodendron maius: genome-wide prediction, expression profiling, and functional characterization of knockout mutants
Source: Front Plant Sci. 2026 Sep 16;17:1924430. doi: 10.3389/fpls.2026.1924430 (PMC13623903; doi:10.3389/fpls.2026.1924430)
Supplement: Supplementary file 1 [file SupplementaryFile1.zip › Supplementary Materials/Supplementary Folder 1.zip!/Oidma1_AssemblyScaffolds_Repeatmasked/knownclusterblast/cluster46/jgi.p_Oidma1_172503_mibig_hits.html]

| MIBiG Protein | Description | MIBiG Cluster | MiBiG Product | % ID | % Coverage | BLAST Score | E-value |
| --- | --- | --- | --- | --- | --- | --- | --- |
| BAN19720.1 | polyketide\_synthase | BGC0001252 | ACR-toxin\_biosynthetic\_gene\_cluster | 50 | 101.472929936 | 2455 | 0.0 |
| EHA28244.1 | hypothetical\_protein | BGC0001143 | Azanigerone\_biosynthetic\_gene\_cluster | 33 | 103.26433121 | 1276 | 0.0 |
| BAC20566.1 | polyketide\_synthase | BGC0000039 | Compactin\_biosynthetic\_gene\_cluster | 33 | 103.901273885 | 1265 | 0.0 |
| AMY15057.1 | tetraketide\_synthase\_MF-SQTKS | BGC0001339 | Squalestatin\_S1\_biosynthetic\_gene\_cluster | 33 | 104.299363057 | 1251 | 0.0 |
| AAD34559.1 | polyketide\_synthase | BGC0000089 | Lovastatin\_biosynthetic\_gene\_cluster | 32 | 103.662420382 | 1194 | 0.0 |
| BAD83684.1 | PKSN\_polyketide\_synthase\_for\_alternapyrone\_biosynthesis | BGC0000012 | Alternapyrone\_biosynthetic\_gene\_cluster | 32 | 103.503184713 | 1159 | 0.0 |
| ABA02240.1 | polyketide\_synthase | BGC0000098 | Monacolin\_K\_biosynthetic\_gene\_cluster | 31 | 103.26433121 | 1151 | 0.0 |
| CAP95405.1 |  | BGC0001404 | Sorbicillin\_biosynthetic\_gene\_cluster | 31 | 105.453821656 | 1148 | 0.0 |
| CBF87072.1 | polyketide\_synthase,\_putative\_(Eurofung) | BGC0001290 | Emericellamide\_A\_/\_emericellamide\_B\_biosynthetic\_gene\_cluster | 32 | 102.866242038 | 1139 | 0.0 |
| EAA65604.1 | hypothetical\_protein | BGC0000022 | Asperfuranone\_biosynthetic\_gene\_cluster | 31 | 105.891719745 | 1115 | 0.0 |
| AAB08104.3 | polyketide\_synthase | BGC0000155\_c3 | T-toxin\_biosynthetic\_gene\_cluster | 32 | 103.941082803 | 1106 | 0.0 |
| ANF07288.1 | hrPKS | BGC0001340 | Byssochlamic\_acid\_biosynthetic\_gene\_cluster | 30 | 104.936305732 | 1074 | 1.79999999998e-313 |
| AAD43562.2 | Fum1p | BGC0000062 | Fumonisin\_biosynthetic\_gene\_cluster | 32 | 105.055732484 | 1063 | 3.3e-310 |
| BAJ09789.1 | polyketide\_synthase | BGC0000146 | Solanapyrone\_biosynthetic\_gene\_cluster | 30 | 98.8455414013 | 942 | 1.1e-273 |
| BAD97694.1 | Aft9-1 | BGC0000003 | AF-toxin\_biosynthetic\_gene\_cluster | 28 | 99.9203821656 | 798 | 2.6e-230 |
| EPE34340.1 | Thiolase-like\_protein | BGC0001035 | Pneumocandin\_biosynthetic\_gene\_cluster | 30 | 86.2261146497 | 779 | 1.6e-224 |
| ACB12550.1 | Fum1 | BGC0000063 | Fumonisin\_biosynthetic\_gene\_cluster | 30 | 86.1464968153 | 760 | 4.6e-219 |
| AFP73394.1 | FusA | BGC0001268 | Fusarin\_biosynthetic\_gene\_cluster | 31 | 66.9585987261 | 712 | 1.9e-204 |
| AAT28740.1 | FUSS | BGC0000064 | Fusarin\_biosynthetic\_gene\_cluster | 31 | 66.4808917197 | 702 | 2e-201 |
| ABA02239.1 | polyketide\_synthase | BGC0000098 | Monacolin\_K\_biosynthetic\_gene\_cluster | 29 | 75.199044586 | 691 | 4.5e-198 |
| EAW09117.1 | hybrid\_NRPS/PKS\_enzyme,\_putative | BGC0000983 | Cytochalasin\_biosynthetic\_gene\_cluster | 31 | 72.7707006369 | 686 | 1.1e-196 |
| BAV32159.1 | polyketide\_synthase | BGC0001373 | Sordarin\_biosynthetic\_gene\_cluster | 34 | 50.3184713376 | 673 | 7.5e-193 |
| ADN43685.1 | DmbS | BGC0001136 | Desmethylbassianin\_biosynthetic\_gene\_cluster | 30 | 73.6464968153 | 671 | 2.8e-192 |
| AAV66110.2 | fusaridione\_A\_synthetase | BGC0000992 | Fusaridione\_A\_biosynthetic\_gene\_cluster | 29 | 76.9506369427 | 666 | 1.2e-190 |
| BAC20564.1 | polyketide\_synthase | BGC0000039 | Compactin\_biosynthetic\_gene\_cluster | 28 | 77.5875796178 | 653 | 1.4e-186 |
| AKC54422.1 | fumosorinone\_biosynthesis\_polyketide\_synthase | BGC0001218 | Fumosorinone\_biosynthetic\_gene\_cluster | 31 | 68.7898089172 | 648 | 2.6e-185 |
| EWG54266.1 | hypothetical\_protein | BGC0001190 | Fusaric\_acid\_biosynthetic\_gene\_cluster | 32 | 51.5525477707 | 639 | 1.6e-182 |
| ACD39767.1 | reducing\_polyketide\_synthase | BGC0000077 | Hypothemycin\_biosynthetic\_gene\_cluster | 35 | 49.2436305732 | 636 | 1e-181 |
| ACD39758.1 | reducing\_polyketide\_synthase | BGC0000076 | Hypothemycin\_biosynthetic\_gene\_cluster | 35 | 49.2436305732 | 636 | 1e-181 |
| AHV78252.1 | ResS1 | BGC0001246 | Trans-resorcylide\_biosynthetic\_gene\_cluster | 34 | 49.800955414 | 634 | 5e-181 |
| AGC95324.1 | CurS1 | BGC0000045 | Dehydrocurvularin\_biosynthetic\_gene\_cluster | 34 | 51.3535031847 | 634 | 5e-181 |
| AHV78245.1 | LasS1 | BGC0001245 | Lasiodiplodin\_biosynthetic\_gene\_cluster | 34 | 50.7563694268 | 626 | 1e-178 |
| EHA19289.1 | hypothetical\_protein | BGC0001124 | Pyranonigrin\_E\_biosynthetic\_gene\_cluster | 34 | 52.1894904459 | 624 | 5.2e-178 |
| ACD39774.1 | reducing\_polyketide\_synthase | BGC0000134 | Radicicol\_biosynthetic\_gene\_cluster | 33 | 49.3630573248 | 619 | 1.3e-176 |
| ABB90283.1 | polyketide\_synthase | BGC0001057 | Zearalenone\_biosynthetic\_gene\_cluster | 34 | 49.1640127389 | 618 | 2.8e-176 |
| ABB76806.1 | polyketide\_synthase\_2 | BGC0000155\_c2 | T-toxin\_biosynthetic\_gene\_cluster | 34 | 48.0891719745 | 617 | 8.3e-176 |
| AGO86662.1 | equisetin\_synthetase | BGC0001255 | Equisetin\_biosynthetic\_gene\_cluster | 30 | 65.8041401274 | 613 | 1.2e-174 |
| CBF80487.1 | hybrid\_PKS-NRPS\_(Eurofung) | BGC0000959 | Aspyridone\_biosynthetic\_gene\_cluster | 31 | 67.8742038217 | 599 | 2.3e-170 |
| AKL78824.1 | GLPKS3 | BGC0001187 | Xenolozoyenone\_biosynthetic\_gene\_cluster | 34 | 49.2436305732 | 592 | 2.9e-168 |
| EAU29808.1 | hypothetical\_protein | BGC0001400 | Citreoviridin\_biosynthetic\_gene\_cluster | 31 | 60.9872611465 | 562 | 2.4e-159 |
| ACZ57548.1 | polyketide\_synthase | BGC0000046 | Depudecin\_biosynthetic\_gene\_cluster | 34 | 46.7356687898 | 558 | 3.5e-158 |
| OAQ83765.1 | KR\_domain-containing\_protein | BGC0001358 | Leucinostatins\_biosynthetic\_gene\_cluster | 29 | 66.3614649682 | 555 | 2.3e-157 |
| EAQ86385.1 | hypothetical\_protein | BGC0001405 | Chaetoviridin\_/\_chaetomugilin\_biosynthetic\_gene\_cluster | 33 | 48.4474522293 | 548 | 3.6e-155 |
| ACS68554.1 | hybrid\_PKS-NRPS\_protein | BGC0001026 | NG-391\_biosynthetic\_gene\_cluster | 33 | 49.1640127389 | 542 | 2.6e-153 |
| AIA58899.1 | HRPKS | BGC0001141 | Brefeldin\_biosynthetic\_gene\_cluster | 34 | 43.8694267516 | 542 | 2.6e-153 |
| CAO91861.1 | putative\_PKS-NRPS\_protein | BGC0000968 | Chaetoglobosin\_biosynthetic\_gene\_cluster | 32 | 50.0398089172 | 536 | 1.9e-151 |
| BAK26562.1 | PKS-NRPS\_hybrid | BGC0000977 | Cyclopiazonic\_acid\_biosynthetic\_gene\_cluster | 34 | 43.6703821656 | 521 | 6.2e-147 |
| EAL85113.2 | hybrid\_PKS-NRPS\_enzyme,\_putative | BGC0001037 | Pseurotin\_A\_biosynthetic\_gene\_cluster | 32 | 50.1194267516 | 515 | 3.4e-145 |
| EAL89230.2 | LovB-like\_polyketide\_synthase,\_putative | BGC0000129 | Pyripyropene\_biosynthetic\_gene\_cluster | 33 | 42.7945859873 | 509 | 1.9e-143 |
| BAJ14522.1 | polyketide\_synthase | BGC0001254 | ACT-Toxin\_II\_biosynthetic\_gene\_cluster | 34 | 42.5557324841 | 509 | 2.4e-143 |
| AMY15068.1 | hexaketide\_synthase\_MF-SQHKS | BGC0001339 | Squalestatin\_S1\_biosynthetic\_gene\_cluster | 31 | 48.7261146497 | 509 | 2.4e-143 |
| ctg1\_orf0002 |  | BGC0001068 | Pyripyropene\_A\_biosynthetic\_gene\_cluster | 32 | 42.5955414013 | 507 | 9.2e-143 |
| BAQ25466.1 | polyketide\_synthase | BGC0001264 | Betaenone\_A\_/\_betaenone\_B\_/\_betaenone\_C\_biosynthetic\_gene\_cluster | 33 | 42.9538216561 | 503 | 1e-141 |
| KGO40478.1 | Acyl\_transferase/acyl\_hydrolase/lysophospholipase | BGC0001205 | Communesin\_biosynthetic\_gene\_cluster | 32 | 48.2085987261 | 503 | 1.7e-141 |
| CBX87032.1 | BcBOA9\_protein | BGC0001243\_c3 | Botryenalol\_/\_botrydial\_/\_dihydrobotrydial\_/\_botryendial\_/ | 32 | 47.1337579618 | 486 | 2.2e-136 |
| EAL85129.1 | polyketide\_synthase,\_putative | BGC0001067 | Fumagillin\_biosynthetic\_gene\_cluster | 31 | 50.2388535032 | 481 | 5.4e-135 |
| AAK57189.1 | MxaE | BGC0001022 | Myxalamid\_biosynthetic\_gene\_cluster | 31 | 46.6958598726 | 474 | 6.6e-133 |
| AIW82279.1 | PuwB | BGC0001125 | Puwainaphycins\_biosynthetic\_gene\_cluster | 30 | 48.8853503185 | 470 | 1.6e-131 |
| CAL69597.1 | nonribosomal\_peptide\_synthase | BGC0001049 | Tenellin\_biosynthetic\_gene\_cluster | 35 | 37.8582802548 | 468 | 4.8e-131 |
| EAU38971.1 | hypothetical\_protein | BGC0001122 | Isoflavipucine\_biosynthetic\_gene\_cluster | 33 | 42.9936305732 | 468 | 6.2e-131 |
| CBD77732.1 | polyketide\_synthase | BGC0000974 | Crocacin\_biosynthetic\_gene\_cluster | 35 | 38.0573248408 | 467 | 1.4e-130 |
| AGC45619.1 | polyketide\_synthase | BGC0001394 | Phenalamide\_biosynthetic\_gene\_cluster | 31 | 46.2977707006 | 465 | 4e-130 |
| ABL86391.1 | hybrid\_polyketide\_synthase\_and\_nonribosomal\_peptide\_synthetase | BGC0000999 | Heat-stable\_antifungal\_factor\_biosynthetic\_gene\_cluster | 31 | 46.3375796178 | 462 | 2.6e-129 |
| OAQ83760.1 | polyketide\_synthase | BGC0001358 | Leucinostatins\_biosynthetic\_gene\_cluster | 28 | 49.601910828 | 462 | 3.4e-129 |
| AAF62884.1 | EpoE | BGC0000991 | Epothilone\_biosynthetic\_gene\_cluster | 31 | 46.6958598726 | 462 | 4.5e-129 |
| ADB12492.1 | EpoE | BGC0000990 | Epothilone\_biosynthetic\_gene\_cluster | 31 | 46.6958598726 | 460 | 9.9e-129 |
| CAO98850.1 | polyketide\_synthase\_AufG | BGC0000023 | Aurafuron\_biosynthetic\_gene\_cluster | 30 | 49.0445859873 | 459 | 2.2e-128 |
| CAQ43076.1 | polyketide\_synthase | BGC0000970 | Chondrochloren\_biosynthetic\_gene\_cluster | 31 | 46.6958598726 | 458 | 4.9e-128 |
| CAO98847.1 | polyketide\_synthase\_AufC | BGC0000023 | Aurafuron\_biosynthetic\_gene\_cluster | 30 | 48.9649681529 | 457 | 1.1e-127 |
| ACB46196.1 | polyketide\_synthase | BGC0000989 | Epothilone\_biosynthetic\_gene\_cluster | 31 | 47.0939490446 | 456 | 1.4e-127 |
| CAJ46690.1 | polyketide\_synthase | BGC0000969 | Chondramide\_biosynthetic\_gene\_cluster | 31 | 48.2484076433 | 455 | 4.2e-127 |
| BAG17643.1 | putative\_NRPS-type-I\_PKS\_fusion\_protein | BGC0001043 | SGR\_PTMs\_biosynthetic\_gene\_cluster | 32 | 45.7006369427 | 452 | 3.5e-126 |
| AAF26922.1 | polyketide\_synthase | BGC0000988 | Epothilone\_biosynthetic\_gene\_cluster | 31 | 46.7356687898 | 451 | 4.6e-126 |
| CAO98879.1 | polyketide\_synthase\_AufD | BGC0000023 | Aurafuron\_biosynthetic\_gene\_cluster | 33 | 37.5398089172 | 451 | 7.9e-126 |
| CAG28678.1 | polyketide\_synthase | BGC0001023 | Myxochromide\_biosynthetic\_gene\_cluster | 30 | 48.5668789809 | 450 | 1e-125 |
| CAD19091.1 | StiG\_protein | BGC0000153 | Stigmatellin\_biosynthetic\_gene\_cluster | 30 | 44.7054140127 | 450 | 1.8e-125 |
| CAQ43075.1 | polyketide\_synthase | BGC0000970 | Chondrochloren\_biosynthetic\_gene\_cluster | 31 | 46.9347133758 | 445 | 5.6e-124 |
| AIT55263.1 | GulF | BGC0000072 | Gulmirecin\_biosynthetic\_gene\_cluster | 30 | 46.2977707006 | 444 | 9.6e-124 |
| AGC45620.1 | polyketide\_synthase | BGC0001394 | Phenalamide\_biosynthetic\_gene\_cluster | 34 | 37.7388535032 | 444 | 9.6e-124 |
| CCE88376.1 | polyketide\_synthase | BGC0001034 | Pellasoren\_biosynthetic\_gene\_cluster | 30 | 48.9649681529 | 443 | 1.6e-123 |
| BAJ16467.1 | polyketide\_synthase | BGC0000058 | FD-891\_biosynthetic\_gene\_cluster | 29 | 48.0891719745 | 440 | 1.1e-122 |
| CAL58684.1 | polyketide\_synthase | BGC0000149 | Spirangien\_biosynthetic\_gene\_cluster | 30 | 47.7707006369 | 438 | 4e-122 |
| EFL02193.1 | amino\_acid\_adenylation\_domain-containing\_protein | BGC0000996 | Frontalamides\_biosynthetic\_gene\_cluster | 30 | 46.0589171975 | 438 | 6.9e-122 |
| AAK19883.1 | soraphen\_polyketide\_synthase\_A | BGC0000147 | Soraphen\_biosynthetic\_gene\_cluster | 30 | 47.2133757962 | 438 | 6.9e-122 |
| ABM21570.1 | CrpB | BGC0000975 | Cryptophycin\_biosynthetic\_gene\_cluster | 28 | 48.6863057325 | 437 | 1.2e-121 |
| BAF02922.1 | type\_I\_polyketide\_synthase | BGC0000073 | Halstoctacosanolide\_biosynthetic\_gene\_cluster | 29 | 48.2882165605 | 435 | 3.4e-121 |
| AAA79984.2 | soraphen\_polyketide\_synthase\_B | BGC0000147 | Soraphen\_biosynthetic\_gene\_cluster | 31 | 46.4570063694 | 433 | 1.7e-120 |
| AEE88284.1 | CurF | BGC0000976 | Curacin\_biosynthetic\_gene\_cluster | 29 | 46.5366242038 | 430 | 1.1e-119 |
| AAK57187.1 | MxaC | BGC0001022 | Myxalamid\_biosynthetic\_gene\_cluster | 32 | 39.8885350318 | 430 | 1.9e-119 |
| AAT70101.1 | CurF | BGC0001165 | Curacin\_biosynthetic\_gene\_cluster | 29 | 46.5366242038 | 428 | 4.2e-119 |
| ADZ24998.1 | polyketide\_synthase | BGC0000380 | Leupyrrin\_biosynthetic\_gene\_cluster | 31 | 46.974522293 | 428 | 4.2e-119 |
| CBD77734.1 | polyketide\_synthase | BGC0000974 | Crocacin\_biosynthetic\_gene\_cluster | 33 | 37.898089172 | 428 | 4.2e-119 |
| CAJ46689.1 | polyketide\_synthase | BGC0000969 | Chondramide\_biosynthetic\_gene\_cluster | 31 | 46.377388535 | 428 | 4.2e-119 |
| ACN64831.1 | PokM1 | BGC0001061 | Polyketomycin\_biosynthetic\_gene\_cluster | 33 | 40.4856687898 | 428 | 7.1e-119 |
| XP\_001220460.1 | hypothetical\_protein | BGC0001182 | Chaetoglobosins\_biosynthetic\_gene\_cluster | 33 | 37.5398089172 | 427 | 9.3e-119 |
| AIT55259.1 | GulB | BGC0000072 | Gulmirecin\_biosynthetic\_gene\_cluster | 33 | 38.1767515924 | 427 | 1.2e-118 |
| AAF19810.1 | MtaB | BGC0001024 | Myxothiazol\_biosynthetic\_gene\_cluster | 31 | 46.2181528662 | 426 | 1.6e-118 |
| CAL58686.1 | polyketide\_synthase | BGC0000149 | Spirangien\_biosynthetic\_gene\_cluster | 31 | 44.0286624204 | 426 | 2.1e-118 |
| AAF19813.1 | MtaE | BGC0001024 | Myxothiazol\_biosynthetic\_gene\_cluster | 32 | 38.6544585987 | 426 | 2.7e-118 |
| AAX98184.1 | polyketide\_synthase\_type\_I | BGC0000052 | ECO-02301\_biosynthetic\_gene\_cluster | 29 | 48.6464968153 | 425 | 4.6e-118 |
| BAH02268.1 | polyketide\_synthase | BGC0000126 | Pladienolide\_biosynthetic\_gene\_cluster | 29 | 48.0095541401 | 424 | 7.9e-118 |
| CAL58685.1 | polyketide\_synthase | BGC0000149 | Spirangien\_biosynthetic\_gene\_cluster | 34 | 37.5796178344 | 423 | 1.8e-117 |
| CAQ43077.1 | polyketide\_synthase | BGC0000970 | Chondrochloren\_biosynthetic\_gene\_cluster | 29 | 50.0 | 423 | 1.8e-117 |
| CAD19089.1 | StiE\_protein | BGC0000153 | Stigmatellin\_biosynthetic\_gene\_cluster | 33 | 35.5095541401 | 423 | 2.3e-117 |
| CUW01175.1 | Type\_I\_polyketide\_synthase | BGC0001359 | PM100117\_/\_PM100118\_biosynthetic\_gene\_cluster | 30 | 48.2882165605 | 423 | 2.3e-117 |
| BAJ16468.1 | polyketide\_synthase | BGC0000058 | FD-891\_biosynthetic\_gene\_cluster | 30 | 49.0047770701 | 423 | 2.3e-117 |
| AHH34189.1 | polyketide\_synthase | BGC0001162 | 1-heptadecene\_biosynthetic\_gene\_cluster | 31 | 40.8837579618 | 423 | 2.3e-117 |
| BAP34733.1 | type\_I\_polyketide\_synthase | BGC0000078 | Incednine\_biosynthetic\_gene\_cluster | 29 | 47.6114649682 | 422 | 3.9e-117 |
| CCE88378.1 | polyketide\_synthase | BGC0001034 | Pellasoren\_biosynthetic\_gene\_cluster | 30 | 46.4570063694 | 421 | 6.7e-117 |
| CAL58682.1 | polyketide\_synthase | BGC0000149 | Spirangien\_biosynthetic\_gene\_cluster | 31 | 41.6799363057 | 421 | 8.7e-117 |
| BAJ16470.1 | polyketide\_synthase | BGC0000058 | FD-891\_biosynthetic\_gene\_cluster | 30 | 46.9347133758 | 419 | 2.5e-116 |
| CAQ34919.1 | polyketide\_synthase | BGC0000986 | Dkxanthene\_biosynthetic\_gene\_cluster | 29 | 49.0047770701 | 419 | 2.5e-116 |
| AAT70104.1 | CurI | BGC0001165 | Curacin\_biosynthetic\_gene\_cluster | 32 | 38.1369426752 | 418 | 4.3e-116 |
| AEE88281.1 | CurI | BGC0000976 | Curacin\_biosynthetic\_gene\_cluster | 32 | 38.1369426752 | 418 | 4.3e-116 |
| AFU82616.1 | polyketide\_synthase | BGC0000998 | Guadinomine\_biosynthetic\_gene\_cluster | 30 | 46.2977707006 | 418 | 4.3e-116 |
| AAX98192.1 | polyketide\_synthase\_type\_I | BGC0000052 | ECO-02301\_biosynthetic\_gene\_cluster | 29 | 48.0493630573 | 418 | 7.4e-116 |
| AEZ64504.1 | Herc | BGC0001065 | Herboxidiene\_biosynthetic\_gene\_cluster | 30 | 46.3375796178 | 418 | 7.4e-116 |
| ACB37755.1 | putative\_type\_I\_polyketide\_synthase | BGC0000162 | Tetrocarcin\_A\_biosynthetic\_gene\_cluster | 29 | 46.8949044586 | 415 | 4.8e-115 |
| AAZ77673.1 | ChlB1 | BGC0000036 | Chlorothricin\_biosynthetic\_gene\_cluster | 32 | 42.4761146497 | 415 | 4.8e-115 |
| ALD82524.1 | polyketide\_synthase | BGC0001212 | Nannocystin\_biosynthetic\_gene\_cluster | 30 | 50.3184713376 | 414 | 8.2e-115 |
| BAH02270.1 | polyketide\_synthase | BGC0000126 | Pladienolide\_biosynthetic\_gene\_cluster | 29 | 48.4872611465 | 413 | 1.4e-114 |
| AAW03325.1 | CtaB | BGC0000982 | Cystothiazole\_A\_biosynthetic\_gene\_cluster | 32 | 38.5350318471 | 413 | 1.4e-114 |
| CAD19088.1 | StiD\_protein | BGC0000153 | Stigmatellin\_biosynthetic\_gene\_cluster | 33 | 39.1321656051 | 413 | 1.4e-114 |
| BAC57028.1 | protomycinolide\_IV\_synthase\_1 | BGC0000102 | Mycinamicin\_biosynthetic\_gene\_cluster | 32 | 36.1863057325 | 413 | 1.4e-114 |
| ADF88276.1 | polyketide\_synthase | BGC0000981 | Cylindrospermopsin\_biosynthetic\_gene\_cluster | 30 | 46.0191082803 | 412 | 3.1e-114 |
| AAT70107.1 | CurL | BGC0001165 | Curacin\_biosynthetic\_gene\_cluster | 29 | 39.928343949 | 412 | 3.1e-114 |
| AEE88278.1 | CurL | BGC0000976 | Curacin\_biosynthetic\_gene\_cluster | 29 | 39.928343949 | 412 | 3.1e-114 |
| BAF02926.1 | type\_I\_polyketide\_synthase | BGC0000073 | Halstoctacosanolide\_biosynthetic\_gene\_cluster | 29 | 48.3678343949 | 412 | 4e-114 |
| CAC22145.1 | putative\_type\_I\_polyketide\_synthase | BGC0000038 | Coelimycin\_biosynthetic\_gene\_cluster | 28 | 52.5079617834 | 411 | 9e-114 |
| AMK48247.1 | 6-methylsalicylic\_acid\_synthase | BGC0001351\_c2 | Cahuitamycins\_biosynthetic\_gene\_cluster | 33 | 40.6449044586 | 411 | 9e-114 |
| CBD77736.1 | polyketide\_synthase | BGC0000974 | Crocacin\_biosynthetic\_gene\_cluster | 29 | 46.5366242038 | 410 | 1.2e-113 |
| BAF02927.1 | type\_I\_polyketide\_synthase | BGC0000073 | Halstoctacosanolide\_biosynthetic\_gene\_cluster | 28 | 48.5270700637 | 410 | 2e-113 |
| BAC68129.1 | modular\_polyketide\_synthase | BGC0000059 | Filipin\_biosynthetic\_gene\_cluster | 29 | 47.7707006369 | 409 | 2.6e-113 |
| AAX98191.1 | polyketide\_synthase\_type\_I | BGC0000052 | ECO-02301\_biosynthetic\_gene\_cluster | 29 | 51.5923566879 | 409 | 3.4e-113 |
| BAF92601.1 | iterative\_type\_I\_PKS | BGC0000118 | Pactamycin\_biosynthetic\_gene\_cluster | 31 | 43.949044586 | 408 | 5.8e-113 |
| ACJ24875.1 | 6-methylsalicylic\_acid\_synthase | BGC0000119 | Pactamycin\_biosynthetic\_gene\_cluster | 31 | 43.949044586 | 408 | 5.8e-113 |
| CUW01176.1 | Type\_I\_polyketide\_synthase | BGC0001359\_c2 | PM100117\_/\_PM100118\_biosynthetic\_gene\_cluster | 29 | 49.3232484076 | 408 | 7.6e-113 |
| AEZ64503.1 | Herd | BGC0001065 | Herboxidiene\_biosynthetic\_gene\_cluster | 30 | 53.7420382166 | 408 | 7.6e-113 |
| AAS98782.1 | JamK | BGC0001001 | Jamaicamide\_biosynthetic\_gene\_cluster | 34 | 33.8375796178 | 408 | 7.6e-113 |
| AAS98781.1 | JamJ | BGC0001001 | Jamaicamide\_biosynthetic\_gene\_cluster | 28 | 46.7356687898 | 407 | 1.3e-112 |
| ABK32256.1 | AmbB | BGC0000014 | Ambruticin\_biosynthetic\_gene\_cluster | 31 | 40.923566879 | 407 | 1.3e-112 |
| BAT51066.1 | type\_I\_polyketide\_synthase | BGC0001296 | Streptazone\_E\_biosynthetic\_gene\_cluster | 28 | 53.5031847134 | 406 | 1.7e-112 |
| CAD89773.1 | MelB\_protein | BGC0001010 | Melithiazol\_biosynthetic\_gene\_cluster | 31 | 38.7738853503 | 406 | 2.2e-112 |
| AHH99926.1 | PKS\_I | BGC0000002 | Aculeximycin\_biosynthetic\_gene\_cluster | 29 | 47.8503184713 | 406 | 2.2e-112 |
| WP\_043476516.1 | beta-ketoacyl\_synthase | BGC0001232 | Leucanicidin\_biosynthetic\_gene\_cluster | 29 | 48.5668789809 | 406 | 2.9e-112 |
| AFP87523.1 | type\_I\_polyketide\_synthase | BGC0001159 | Marinopyrrole\_biosynthetic\_gene\_cluster | 31 | 40.127388535 | 406 | 2.9e-112 |
| CAJ88187.2 | putative\_type\_I\_modular\_polyketide\_synthase | BGC0000151 | Stambomycin\_biosynthetic\_gene\_cluster | 29 | 48.6066878981 | 405 | 4.9e-112 |
| ADH04641.1 | TgaC | BGC0001051 | Thuggacin\_biosynthetic\_gene\_cluster | 29 | 48.5668789809 | 405 | 4.9e-112 |
| ABX60152.1 | polyketide\_synthase | BGC0000978 | Cylindrospermopsin\_biosynthetic\_gene\_cluster | 30 | 46.0191082803 | 404 | 8.4e-112 |
| AAZ77696.1 | ChlA3 | BGC0000036 | Chlorothricin\_biosynthetic\_gene\_cluster | 30 | 46.6560509554 | 404 | 8.4e-112 |
| BAC76492.1 | lankamycin\_synthase\_LkmAII | BGC0000085 | Lankamycin\_biosynthetic\_gene\_cluster | 30 | 46.4968152866 | 404 | 1.1e-111 |
| AFL48525.1 | laidlomycin\_polyketide\_synthase\_(loading\_module\_and\_module\_1) | BGC0000084 | Laidlomycin\_biosynthetic\_gene\_cluster | 29 | 47.6512738854 | 403 | 1.4e-111 |
| CQR60493.1 | Polyketide\_synthase,\_type\_I,\_modules:\_9\_and\_10 | BGC0001287 | Chaxamycin\_A\_/\_chaxamycin\_B\_/\_chaxamycin\_C\_/\_chaxamycin\_D | 29 | 46.4570063694 | 403 | 1.9e-111 |
| WP\_042799407.1 | polyketide\_synthase | BGC0001283 | Arsenopolyketides\_biosynthetic\_gene\_cluster | 32 | 42.8343949045 | 403 | 1.9e-111 |
| ABK32291.1 | JerE | BGC0000080 | Jerangolid\_biosynthetic\_gene\_cluster | 32 | 38.5748407643 | 403 | 2.5e-111 |
| AAK73502.1 | AmphJ | BGC0000015 | Amphotericin\_biosynthetic\_gene\_cluster | 30 | 47.2133757962 | 402 | 3.2e-111 |
| ADC79618.1 | BafAIII | BGC0000028 | Bafilomycin\_biosynthetic\_gene\_cluster | 29 | 48.6464968153 | 402 | 4.2e-111 |
| AAQ84156.1 | Plm1 | BGC0000123 | Phoslactomycin\_B\_biosynthetic\_gene\_cluster | 29 | 51.0748407643 | 402 | 4.2e-111 |
| ABK32259.1 | AmbE | BGC0000014 | Ambruticin\_biosynthetic\_gene\_cluster | 29 | 46.6560509554 | 401 | 5.5e-111 |
| BAF85843.1 | modular\_polyketide\_synthase | BGC0000109 | Nemadectin\_biosynthetic\_gene\_cluster | 28 | 47.8503184713 | 401 | 7.1e-111 |
| CAQ18829.1 | polyketide\_synthase | BGC0000954 | Ajudazol\_biosynthetic\_gene\_cluster | 32 | 38.1369426752 | 401 | 7.1e-111 |
| AAS98777.1 | JamE | BGC0001001 | Jamaicamide\_biosynthetic\_gene\_cluster | 31 | 39.0127388535 | 401 | 9.3e-111 |
| AAW03328.1 | CtaE | BGC0000982 | Cystothiazole\_A\_biosynthetic\_gene\_cluster | 32 | 37.9777070064 | 400 | 1.6e-110 |
| AAZ94389.1 | modular\_polyketide\_synthase | BGC0000040 | Concanamycin\_A\_biosynthetic\_gene\_cluster | 29 | 45.9394904459 | 400 | 1.6e-110 |
| AAF71767.1 | NysJ | BGC0000115 | Nystatin\_biosynthetic\_gene\_cluster | 30 | 46.2977707006 | 399 | 2.1e-110 |
| AFL48529.1 | laidlomycin\_polyketide\_synthase\_(module\_5\_and\_module\_6) | BGC0000084 | Laidlomycin\_biosynthetic\_gene\_cluster | 31 | 48.6464968153 | 399 | 2.1e-110 |
| AAP42874.1 | NanA8 | BGC0000105 | Nanchangmycin\_biosynthetic\_gene\_cluster | 30 | 48.8057324841 | 399 | 2.1e-110 |
| BAD08360.1 | polyketide\_synthase\_modules\_7-8 | BGC0000167 | Vicenistatin\_biosynthetic\_gene\_cluster | 29 | 48.0891719745 | 399 | 2.1e-110 |
| BAO98805.1 | putative\_polyketide\_synthase | BGC0001002 | Jawsamycin\_biosynthetic\_gene\_cluster | 30 | 47.173566879 | 399 | 2.7e-110 |
| BAF85844.1 | modular\_polyketide\_synthase | BGC0000109 | Nemadectin\_biosynthetic\_gene\_cluster | 31 | 47.2133757962 | 399 | 3.5e-110 |
| ABI94380.1 | tautomycetin\_biosynthetic\_PKS | BGC0000157 | Tautomycetin\_biosynthetic\_gene\_cluster | 29 | 47.5318471338 | 399 | 3.5e-110 |
| AEZ53950.1 | polyketide\_synthase | BGC0000144 | Salinomycin\_biosynthetic\_gene\_cluster | 27 | 53.1050955414 | 399 | 3.5e-110 |
| AKD43522.1 | Type\_I\_polyketide\_synthase | BGC0001409 | Dutomycin\_biosynthetic\_gene\_cluster | 29 | 46.2977707006 | 398 | 4.6e-110 |
| CUW01171.1 | Type\_I\_polyketide\_synthase | BGC0001359 | PM100117\_/\_PM100118\_biosynthetic\_gene\_cluster | 31 | 37.8184713376 | 398 | 4.6e-110 |
| BAP34763.1 | type\_I\_polyketide\_synthase | BGC0000078 | Incednine\_biosynthetic\_gene\_cluster | 30 | 48.4872611465 | 398 | 6e-110 |
| ABY66019.1 | 6-methylsalicylic\_acid\_synthase | BGC0001008 | Maduropeptin\_biosynthetic\_gene\_cluster | 34 | 35.4299363057 | 398 | 6e-110 |
| ABB05104.1 | LipPks3 | BGC0001003 | Lipomycin\_biosynthetic\_gene\_cluster | 29 | 48.8455414013 | 398 | 6e-110 |
| ABK32289.1 | JerC | BGC0000080 | Jerangolid\_biosynthetic\_gene\_cluster | 30 | 41.7993630573 | 398 | 7.9e-110 |
| AAY42396.1 | HctD | BGC0001000 | Hectochlorin\_biosynthetic\_gene\_cluster | 31 | 38.4156050955 | 398 | 7.9e-110 |
| BAT51065.1 | type\_I\_polyketide\_synthase | BGC0001296 | Streptazone\_E\_biosynthetic\_gene\_cluster | 29 | 46.576433121 | 398 | 7.9e-110 |
| ADC45538.1 | modular\_polyketide\_synthase | BGC0000093 | Meilingmycin\_biosynthetic\_gene\_cluster | 29 | 47.2133757962 | 398 | 7.9e-110 |
| AFV96138.1 | polyketide\_synthase | BGC0001064 | Cylindrocyclophane\_biosynthetic\_gene\_cluster | 32 | 38.097133758 | 397 | 1e-109 |
| ABK32257.1 | AmbC | BGC0000014 | Ambruticin\_biosynthetic\_gene\_cluster | 29 | 48.4076433121 | 397 | 1.3e-109 |
| BAK64650.1 | polyketide\_synthase | BGC0000135 | Reveromycin\_biosynthetic\_gene\_cluster | 29 | 48.7659235669 | 396 | 2.3e-109 |
| AEP40936.1 | polyketide\_synthase\_type\_I | BGC0000021 | Apoptolidin\_biosynthetic\_gene\_cluster | 30 | 47.8105095541 | 396 | 2.3e-109 |
| ABC84458.1 | NigAIII | BGC0000114 | Nigericin\_biosynthetic\_gene\_cluster | 29 | 46.4570063694 | 396 | 2.3e-109 |
| ACR50775.1 | polyketide\_synthase | BGC0000163 | Tetronasin\_biosynthetic\_gene\_cluster | 29 | 48.4076433121 | 395 | 5.1e-109 |
| CAD89776.1 | MelE\_protein | BGC0001009 | Melithiazol\_biosynthetic\_gene\_cluster | 31 | 38.4952229299 | 395 | 5.1e-109 |
| ADB12491.1 | EpoD | BGC0000990 | Epothilone\_biosynthetic\_gene\_cluster | 31 | 44.6656050955 | 395 | 5.1e-109 |
| AEZ54379.1 | PieA6 | BGC0000124 | Piericidin\_A1\_biosynthetic\_gene\_cluster | 33 | 34.8328025478 | 395 | 5.1e-109 |
| ABV91287.1 | type\_I\_modular\_polyketide\_synthase | BGC0000158 | Tautomycetin\_biosynthetic\_gene\_cluster | 29 | 47.5318471338 | 395 | 5.1e-109 |
| AAM77986.1 | iterative\_type\_I\_polyketide\_synthase | BGC0000112 | Neocarzinostatin\_biosynthetic\_gene\_cluster | 32 | 41.0828025478 | 395 | 5.1e-109 |
| BAK64637.1 | polyketide\_synthase | BGC0000135 | Reveromycin\_biosynthetic\_gene\_cluster | 28 | 48.4474522293 | 395 | 5.1e-109 |
| CAE46851.1 | Type\_I\_modular\_polyketide\_synthase | BGC0000103 | Mycolactone\_biosynthetic\_gene\_cluster | 29 | 42.3964968153 | 394 | 6.7e-109 |
| ABI94379.1 | tautomycetin\_biosynthetic\_PKS | BGC0000157 | Tautomycetin\_biosynthetic\_gene\_cluster | 29 | 45.9394904459 | 394 | 8.7e-109 |
| ADH04660.1 | TugD | BGC0001342 | Thuggacin\_biosynthetic\_gene\_cluster | 33 | 34.9920382166 | 394 | 1.1e-108 |
| AAX98189.1 | polyketide\_synthase\_type\_I | BGC0000052 | ECO-02301\_biosynthetic\_gene\_cluster | 29 | 48.2882165605 | 394 | 1.1e-108 |
| AAG23266.1 | polyketide\_synthase\_extender\_modules\_3-4 | BGC0000148 | Spinosad\_biosynthetic\_gene\_cluster | 31 | 38.1767515924 | 394 | 1.1e-108 |
| AAO65799.1 | monensin\_polyketide\_synthase\_modules\_5\_and\_6 | BGC0000100 | Monensin\_biosynthetic\_gene\_cluster | 30 | 47.9697452229 | 394 | 1.1e-108 |
| CAD19092.1 | StiH\_protein | BGC0000153 | Stigmatellin\_biosynthetic\_gene\_cluster | 31 | 38.1767515924 | 393 | 1.5e-108 |
| AAF62883.1 | epoD | BGC0000991 | Epothilone\_biosynthetic\_gene\_cluster | 31 | 44.2675159236 | 393 | 1.5e-108 |
| ACB46195.1 | polyketide\_synthase | BGC0000989 | Epothilone\_biosynthetic\_gene\_cluster | 30 | 44.2277070064 | 393 | 1.5e-108 |
| AAP42859.1 | NanA5 | BGC0000105 | Nanchangmycin\_biosynthetic\_gene\_cluster | 29 | 46.2181528662 | 393 | 1.9e-108 |
| AFV30248.1 | polyketide\_synthase | BGC0000075 | Hygrocin\_biosynthetic\_gene\_cluster | 29 | 49.0843949045 | 393 | 2.5e-108 |
| AAG23263.1 | polyketide\_synthase\_extender\_modules\_5-7 | BGC0000148 | Spinosad\_biosynthetic\_gene\_cluster | 29 | 47.2531847134 | 393 | 2.5e-108 |
| AEZ64505.1 | Herb | BGC0001065 | Herboxidiene\_biosynthetic\_gene\_cluster | 30 | 47.8901273885 | 393 | 2.5e-108 |
| BAO66528.1 | type\_I\_polyketide\_synthase | BGC0000042 | Cremimycin\_biosynthetic\_gene\_cluster | 31 | 40.724522293 | 393 | 2.5e-108 |
| BAO66529.1 | type\_I\_polyketide\_synthase | BGC0000042 | Cremimycin\_biosynthetic\_gene\_cluster | 30 | 47.6910828025 | 393 | 2.5e-108 |
| AAG13919.1 | megalomicin\_6-deoxyerythronolide\_B\_synthase\_3 | BGC0000092 | Megalomicin\_biosynthetic\_gene\_cluster | 32 | 34.9124203822 | 393 | 2.5e-108 |
| BAH02269.1 | polyketide\_synthase | BGC0000126 | Pladienolide\_biosynthetic\_gene\_cluster | 28 | 54.2993630573 | 393 | 2.5e-108 |
| AEC13069.1 | fosC | BGC0000060 | Fostriecin\_biosynthetic\_gene\_cluster | 30 | 47.8105095541 | 392 | 3.3e-108 |
| ABB88522.1 | polyketide\_synthase\_type\_I | BGC0000050 | E-837\_biosynthetic\_gene\_cluster | 30 | 49.2834394904 | 392 | 4.3e-108 |
| ABV97155.1 | Acyl\_transferase | BGC0000137 | Rifamycin\_biosynthetic\_gene\_cluster | 30 | 46.6162420382 | 391 | 5.7e-108 |
| CAD55506.1 | putative\_type\_I\_polyketide\_synthase | BGC0000038 | Coelimycin\_biosynthetic\_gene\_cluster | 30 | 46.4968152866 | 391 | 7.4e-108 |
| ABV83230.1 | CppC | BGC0000116 | Nystatin-like\_Pseudonocardia\_polyene\_biosynthetic\_gene\_cluster | 30 | 48.6066878981 | 391 | 7.4e-108 |
| AEZ53949.1 | polyketide\_synthase | BGC0000144 | Salinomycin\_biosynthetic\_gene\_cluster | 28 | 46.7356687898 | 391 | 9.7e-108 |
| AKD43769.1 | HerA2 | BGC0001349 | Heronamide\_biosynthetic\_gene\_cluster | 31 | 41.2818471338 | 390 | 1.3e-107 |
| ABK32288.1 | JerB | BGC0000080 | Jerangolid\_biosynthetic\_gene\_cluster | 31 | 38.2165605096 | 390 | 1.6e-107 |
| AAF26921.1 | polyketide\_synthase | BGC0000988 | Epothilone\_biosynthetic\_gene\_cluster | 30 | 44.2277070064 | 389 | 2.2e-107 |
| ADH04659.1 | TugC | BGC0001342 | Thuggacin\_biosynthetic\_gene\_cluster | 30 | 43.5907643312 | 389 | 2.2e-107 |
| BAQ25511.1 | type\_I\_polyketide\_synthase | BGC0001288 | Maklamicin\_biosynthetic\_gene\_cluster | 33 | 33.877388535 | 389 | 2.2e-107 |
| AAX98186.1 | polyketide\_synthase\_type\_I | BGC0000052 | ECO-02301\_biosynthetic\_gene\_cluster | 28 | 47.173566879 | 389 | 2.2e-107 |
| ADC79616.1 | BafAI | BGC0000028 | Bafilomycin\_biosynthetic\_gene\_cluster | 31 | 37.3407643312 | 389 | 2.8e-107 |
| AAF26919.1 | polyketide\_synthase | BGC0000988 | Epothilone\_biosynthetic\_gene\_cluster | 33 | 37.9777070064 | 389 | 2.8e-107 |
| CAE46843.1 | Type\_I\_modular\_polyketide\_synthase | BGC0000103 | Mycolactone\_biosynthetic\_gene\_cluster | 29 | 45.5812101911 | 389 | 2.8e-107 |
| CAD89777.1 | MelF\_protein | BGC0001009 | Melithiazol\_biosynthetic\_gene\_cluster | 30 | 38.0175159236 | 389 | 3.7e-107 |
| AAB66506.1 | tylactone\_synthase\_modules\_4\_&\_5 | BGC0000166 | Tylactone\_biosynthetic\_gene\_cluster | 30 | 43.4315286624 | 389 | 3.7e-107 |
| ADB12488.1 | EpoA | BGC0000990 | Epothilone\_biosynthetic\_gene\_cluster | 33 | 38.0573248408 | 388 | 4.8e-107 |
| BAO66519.1 | type\_I\_polyketide\_synthase | BGC0000042 | Cremimycin\_biosynthetic\_gene\_cluster | 34 | 34.0366242038 | 388 | 4.8e-107 |
| AAF62880.1 | EpoA | BGC0000991 | Epothilone\_biosynthetic\_gene\_cluster | 33 | 38.0573248408 | 388 | 4.8e-107 |
| ABC84469.1 | NigAIX | BGC0000114 | Nigericin\_biosynthetic\_gene\_cluster | 28 | 47.372611465 | 388 | 4.8e-107 |
| AEZ53945.1 | polyketide\_synthase | BGC0000144 | Salinomycin\_biosynthetic\_gene\_cluster | 29 | 46.4171974522 | 388 | 8.2e-107 |
| ABP55210.1 | beta-ketoacyl\_synthase | BGC0000142 | Salinilactam\_biosynthetic\_gene\_cluster | 30 | 48.5270700637 | 388 | 8.2e-107 |
| EGJ35088.1 | polyketide\_synthase\_module | BGC0001163 | 1-heptadecene\_biosynthetic\_gene\_cluster | 30 | 41.2022292994 | 387 | 1.4e-106 |
| ABV91286.1 | type\_I\_modular\_polyketide\_synthase | BGC0000158 | Tautomycetin\_biosynthetic\_gene\_cluster | 29 | 45.9792993631 | 387 | 1.4e-106 |
| CCP20047.1 | divK\_protein | BGC0001119 | Divergolide\_biosynthetic\_gene\_cluster | 32 | 38.1369426752 | 386 | 1.8e-106 |
| CAQ64686.1 | lasalocid\_modular\_polyketide\_synthase | BGC0000087 | Lasalocid\_biosynthetic\_gene\_cluster | 29 | 48.3280254777 | 386 | 1.8e-106 |
| BAG85026.1 | putative\_polyketide\_synthase | BGC0000086 | Lasalocid\_biosynthetic\_gene\_cluster | 29 | 48.3280254777 | 386 | 1.8e-106 |
| AAT70096.1 | CurA | BGC0001165 | Curacin\_biosynthetic\_gene\_cluster | 30 | 38.4156050955 | 386 | 1.8e-106 |
| AAQ82566.1 | FscF | BGC0000061 | FR-008\_biosynthetic\_gene\_cluster | 31 | 40.8041401274 | 386 | 1.8e-106 |
| AEE88289.1 | CurA | BGC0000976 | Curacin\_biosynthetic\_gene\_cluster | 30 | 38.4156050955 | 386 | 1.8e-106 |
| AAQ84145.1 | Plm5 | BGC0000123 | Phoslactomycin\_B\_biosynthetic\_gene\_cluster | 29 | 48.4872611465 | 386 | 1.8e-106 |
| AAF71774.1 | NysA | BGC0000115 | Nystatin\_biosynthetic\_gene\_cluster | 29 | 45.7404458599 | 386 | 1.8e-106 |
| BAQ25512.1 | type\_I\_polyketide\_synthase | BGC0001288 | Maklamicin\_biosynthetic\_gene\_cluster | 29 | 49.0445859873 | 386 | 3.1e-106 |
| AKD43768.1 | HerA1 | BGC0001349 | Heronamide\_biosynthetic\_gene\_cluster | 28 | 47.6114649682 | 385 | 4.1e-106 |
| AAC69329.1 | type\_I\_polyketide\_synthase\_PikAI | BGC0000094 | Methymycin\_/\_pikromycin\_biosynthetic\_gene\_cluster | 32 | 38.1767515924 | 385 | 4.1e-106 |
| AAD03047.1 | type\_I\_polyketide\_synthase | BGC0000041 | Coronafacic\_acid\_biosynthetic\_gene\_cluster | 29 | 47.372611465 | 385 | 4.1e-106 |
| CAJ88176.1 | putative\_polyketide\_synthase\_B | BGC0000151 | Stambomycin\_biosynthetic\_gene\_cluster | 29 | 46.7356687898 | 385 | 4.1e-106 |
| AAP42855.1 | NanA1 | BGC0000105 | Nanchangmycin\_biosynthetic\_gene\_cluster | 29 | 47.8105095541 | 385 | 4.1e-106 |
| CBD77738.1 | polyketide\_synthase | BGC0000974 | Crocacin\_biosynthetic\_gene\_cluster | 33 | 36.7436305732 | 385 | 5.3e-106 |
| AAX98188.1 | polyketide\_synthase\_type\_I | BGC0000052 | ECO-02301\_biosynthetic\_gene\_cluster | 32 | 35.1114649682 | 385 | 5.3e-106 |
| ACB46192.1 | polyketide\_synthase | BGC0000989 | Epothilone\_biosynthetic\_gene\_cluster | 33 | 37.9777070064 | 384 | 6.9e-106 |
| AEE88282.1 | CurH | BGC0000976 | Curacin\_biosynthetic\_gene\_cluster | 28 | 47.173566879 | 384 | 6.9e-106 |
| AAT70103.1 | CurH | BGC0001165 | Curacin\_biosynthetic\_gene\_cluster | 28 | 47.173566879 | 384 | 6.9e-106 |
| ALD82523.1 | polyketide\_synthase | BGC0001212 | Nannocystin\_biosynthetic\_gene\_cluster | 32 | 40.0079617834 | 384 | 6.9e-106 |
| AAZ94387.1 | modular\_polyketide\_synthase | BGC0000040 | Concanamycin\_A\_biosynthetic\_gene\_cluster | 32 | 36.2659235669 | 384 | 9e-106 |
| BAE93730.1 | type\_I\_polyketide\_synthase | BGC0000164 | Tetronomycin\_biosynthetic\_gene\_cluster | 28 | 46.974522293 | 384 | 9e-106 |
| ctg1\_orf30 |  | BGC0000096 | Midecamycin\_biosynthetic\_gene\_cluster | 32 | 36.9824840764 | 384 | 1.2e-105 |
| CAO85893.1 | modular\_polyketide\_synthase\_NorA | BGC0000110 | Neoaureothin\_biosynthetic\_gene\_cluster | 29 | 47.3328025478 | 384 | 1.2e-105 |
| BAR73019.1 | putative\_PKS\_(KS-AT-DH-KR-ACP-TE) | BGC0001194 | Hitachimycin\_biosynthetic\_gene\_cluster | 30 | 45.9792993631 | 383 | 1.5e-105 |
| BAF02921.1 | type\_I\_polyketide\_synthase | BGC0000073 | Halstoctacosanolide\_biosynthetic\_gene\_cluster | 31 | 38.3359872611 | 383 | 1.5e-105 |
| CAM00065.1 | EryAIII\_Erythromycin\_polyketide\_synthase\_modules\_5\_and\_6 | BGC0000055 | Erythromycin\_biosynthetic\_gene\_cluster | 32 | 34.9522292994 | 383 | 1.5e-105 |
| BAJ16469.1 | polyketide\_synthase | BGC0000058 | FD-891\_biosynthetic\_gene\_cluster | 28 | 46.775477707 | 383 | 1.5e-105 |
| BAB69194.1 | modular\_polyketide\_synthase | BGC0000117 | Oligomycin\_biosynthetic\_gene\_cluster | 28 | 45.9792993631 | 383 | 2e-105 |
| ACR50773.1 | polyketide\_synthase | BGC0000163 | Tetronasin\_biosynthetic\_gene\_cluster | 29 | 47.9697452229 | 383 | 2e-105 |
| AAZ77693.1 | ChlA1 | BGC0000036 | Chlorothricin\_biosynthetic\_gene\_cluster | 32 | 37.3407643312 | 382 | 3.4e-105 |
| AAW03329.1 | CtaF | BGC0000982 | Cystothiazole\_A\_biosynthetic\_gene\_cluster | 31 | 37.8582802548 | 382 | 3.4e-105 |
| AJW65410.1 | type\_I\_modular\_polyketide\_synthase | BGC0001195 | Nocardiopsin\_biosynthetic\_gene\_cluster | 29 | 46.576433121 | 382 | 3.4e-105 |
| CAJ88186.1 | putative\_modular\_polyketide\_synthase | BGC0000151 | Stambomycin\_biosynthetic\_gene\_cluster | 28 | 47.6512738854 | 382 | 4.5e-105 |
| AIT55264.1 | GulA | BGC0000072 | Gulmirecin\_biosynthetic\_gene\_cluster | 32 | 36.9426751592 | 382 | 4.5e-105 |
| BAD08373.1 | polyketide\_synthase\_modules\_1-3 | BGC0000167 | Vicenistatin\_biosynthetic\_gene\_cluster | 28 | 46.6560509554 | 382 | 4.5e-105 |
| AAG23262.1 | polyketide\_synthase\_extender\_modules\_8-10 | BGC0000148 | Spinosad\_biosynthetic\_gene\_cluster | 28 | 48.3678343949 | 382 | 4.5e-105 |
| AFL48532.1 | laidlomycin\_polyketide\_synthase\_(module\_11\_and\_module\_12) | BGC0000084 | Laidlomycin\_biosynthetic\_gene\_cluster | 30 | 41.0828025478 | 382 | 4.5e-105 |
| ACO94468.1 | polyketide\_synthase\_type\_I | BGC0000029 | BE-14106\_biosynthetic\_gene\_cluster | 29 | 46.775477707 | 381 | 5.9e-105 |
| AFP87524.1 | type\_I\_polyketide\_synthase | BGC0001159 | Marinopyrrole\_biosynthetic\_gene\_cluster | 32 | 38.0175159236 | 381 | 7.7e-105 |
| AAF26923.1 | polyketide\_synthase | BGC0000988 | Epothilone\_biosynthetic\_gene\_cluster | 30 | 46.6560509554 | 381 | 1e-104 |
| BAD08358.1 | polyketide\_synthase\_modules\_4 | BGC0000167 | Vicenistatin\_biosynthetic\_gene\_cluster | 28 | 48.3678343949 | 381 | 1e-104 |
| ACB46197.1 | polyketide\_synthase | BGC0000989 | Epothilone\_biosynthetic\_gene\_cluster | 30 | 49.402866242 | 381 | 1e-104 |
| BAD38874.1 | polyketide\_synthase | BGC0000111 | Neocarzilin\_biosynthetic\_gene\_cluster | 30 | 47.8901273885 | 381 | 1e-104 |
| ACO94488.1 | polyketide\_synthase\_type\_I | BGC0000097 | ML-449\_biosynthetic\_gene\_cluster | 29 | 46.7356687898 | 381 | 1e-104 |
| AAF62885.1 | EpoF | BGC0000991 | Epothilone\_biosynthetic\_gene\_cluster | 29 | 48.2882165605 | 380 | 1.3e-104 |
| ACB46485.1 | polyketide\_synthase | BGC0000082 | Kijanimicin\_biosynthetic\_gene\_cluster | 32 | 37.0621019108 | 380 | 1.3e-104 |
| AKD43764.1 | HerF | BGC0001349 | Heronamide\_biosynthetic\_gene\_cluster | 28 | 51.5525477707 | 380 | 1.3e-104 |
| ACF35447.1 | MbcAIII | BGC0000090 | Macbecin\_biosynthetic\_gene\_cluster | 28 | 47.1337579618 | 380 | 1.7e-104 |
| AAX98185.1 | polyketide\_synthase\_type\_I | BGC0000052 | ECO-02301\_biosynthetic\_gene\_cluster | 29 | 46.9347133758 | 380 | 1.7e-104 |
| BAC68125.1 | modular\_polyketide\_synthase | BGC0000059 | Filipin\_biosynthetic\_gene\_cluster | 31 | 36.9824840764 | 379 | 2.2e-104 |
| BAK64649.1 | polyketide\_synthase | BGC0000135 | Reveromycin\_biosynthetic\_gene\_cluster | 32 | 35.5891719745 | 379 | 2.9e-104 |
| ADC45535.1 | modular\_polyketide\_synthase | BGC0000093 | Meilingmycin\_biosynthetic\_gene\_cluster | 33 | 33.877388535 | 379 | 2.9e-104 |
| AAS79460.1 | polyketide\_synthase\_subunit | BGC0000035 | Chalcomycin\_biosynthetic\_gene\_cluster | 29 | 47.5318471338 | 379 | 3.8e-104 |
| CUW01172.1 | Type\_I\_polyketide\_synthase | BGC0001359 | PM100117\_/\_PM100118\_biosynthetic\_gene\_cluster | 31 | 37.4601910828 | 379 | 3.8e-104 |
| WP\_048832936.1 | polyketide\_synthase | BGC0001348 | JBIR-100\_biosynthetic\_gene\_cluster | 28 | 46.5366242038 | 379 | 3.8e-104 |
| ACY06290.1 | type\_I\_polyketide\_synthase | BGC0001042 | Sanglifehrin\_A\_biosynthetic\_gene\_cluster | 32 | 36.9824840764 | 378 | 5e-104 |
| AAO65797.1 | monensin\_polyketide\_synthase\_module\_2 | BGC0000100 | Monensin\_biosynthetic\_gene\_cluster | 28 | 47.8105095541 | 378 | 5e-104 |
| ABK32258.1 | AmbD | BGC0000014 | Ambruticin\_biosynthetic\_gene\_cluster | 28 | 48.7261146497 | 378 | 5e-104 |
| ABK32290.1 | JerD | BGC0000080 | Jerangolid\_biosynthetic\_gene\_cluster | 28 | 48.1687898089 | 378 | 6.5e-104 |
| AAQ84157.1 | Plm2-3 | BGC0000123 | Phoslactomycin\_B\_biosynthetic\_gene\_cluster | 30 | 46.178343949 | 378 | 6.5e-104 |
| AAU93806.2 | polyketide\_synthase\_modules\_3\_and\_4 | BGC0000054 | Erythromycin\_biosynthetic\_gene\_cluster | 32 | 37.0222929936 | 378 | 8.5e-104 |
| CAD19086.1 | StiB\_protein | BGC0000153 | Stigmatellin\_biosynthetic\_gene\_cluster | 30 | 39.0923566879 | 378 | 8.5e-104 |
| BAR73021.1 | putative\_PKS\_(KS-AT-KR-ACP) | BGC0001194 | Hitachimycin\_biosynthetic\_gene\_cluster | 31 | 38.7738853503 | 378 | 8.5e-104 |
| BAB69199.1 | modular\_polyketide\_synthase | BGC0000117 | Oligomycin\_biosynthetic\_gene\_cluster | 30 | 41.4808917197 | 378 | 8.5e-104 |
| AAR16521.1 | RimA | BGC0000138 | Rimocidin\_biosynthetic\_gene\_cluster | 32 | 37.0621019108 | 377 | 1.1e-103 |
| BI06\_RS39075 | polyketide\_synthase | BGC0001232 | Leucanicidin\_biosynthetic\_gene\_cluster | 31 | 36.7436305732 | 377 | 1.1e-103 |
| BAB69198.1 | modular\_polyketide\_synthase | BGC0000117 | Oligomycin\_biosynthetic\_gene\_cluster | 27 | 46.2977707006 | 377 | 1.1e-103 |
| ctg1\_orf31 |  | BGC0000096 | Midecamycin\_biosynthetic\_gene\_cluster | 31 | 37.3805732484 | 377 | 1.1e-103 |
| AGI99494.1 | Type\_I\_polyketide\_synthase | BGC0001004 | Lobophorin\_biosynthetic\_gene\_cluster | 32 | 36.7436305732 | 377 | 1.4e-103 |
| ADH04640.1 | TgaB | BGC0001051 | Thuggacin\_biosynthetic\_gene\_cluster | 30 | 44.6257961783 | 377 | 1.4e-103 |
| AAO06918.1 | GdmAIII | BGC0000066 | Geldanamycin\_biosynthetic\_gene\_cluster | 28 | 47.7707006369 | 377 | 1.4e-103 |
| CAA60460.1 | polyketide\_synthase | BGC0001040 | Rapamycin\_biosynthetic\_gene\_cluster | 29 | 46.1385350318 | 376 | 1.9e-103 |
| ABB86410.1 | GelC | BGC0000067 | Geldanamycin\_biosynthetic\_gene\_cluster | 28 | 47.7707006369 | 376 | 2.5e-103 |
| BAC76491.1 | lankamycin\_synthase\_LkmAIII | BGC0000085 | Lankamycin\_biosynthetic\_gene\_cluster | 32 | 35.071656051 | 376 | 2.5e-103 |
| ABC84470.1 | NIGAVIII | BGC0000114 | Nigericin\_biosynthetic\_gene\_cluster | 29 | 46.8550955414 | 376 | 2.5e-103 |
| CAQ52626.1 | type\_I\_polyketide\_synthase,\_loading\_module\_and\_modules\_1-3 | BGC0001066 | Kendomycin\_biosynthetic\_gene\_cluster | 32 | 38.5748407643 | 376 | 2.5e-103 |
| AAM81585.1 | putative\_type\_I\_polyketide\_synthase | BGC0000047 | Dihydrochalcomycin\_biosynthetic\_gene\_cluster | 28 | 47.7308917197 | 376 | 3.2e-103 |
| CAC20930.1 | PimS0\_protein | BGC0000125 | Pimaricin\_biosynthetic\_gene\_cluster | 32 | 37.4601910828 | 375 | 5.5e-103 |
| AAG23264.1 | polyketide\_synthase\_loading\_and\_extender\_module\_1 | BGC0000148 | Spinosad\_biosynthetic\_gene\_cluster | 31 | 35.4299363057 | 375 | 5.5e-103 |
| AFL48526.1 | laidlomycin\_polyketide\_synthase\_(module\_2) | BGC0000084 | Laidlomycin\_biosynthetic\_gene\_cluster | 29 | 46.4171974522 | 375 | 5.5e-103 |
| ACB46488.1 | polyketide\_synthase | BGC0000082 | Kijanimicin\_biosynthetic\_gene\_cluster | 28 | 47.8503184713 | 374 | 7.2e-103 |
| AGC09487.1 | LobS5 | BGC0001183 | Lobophorin\_biosynthetic\_gene\_cluster | 32 | 36.6242038217 | 374 | 7.2e-103 |
| BAQ25513.1 | type\_I\_polyketide\_synthase | BGC0001288 | Maklamicin\_biosynthetic\_gene\_cluster | 29 | 41.5605095541 | 374 | 7.2e-103 |
| AAK73501.1 | AmphI | BGC0000015 | Amphotericin\_biosynthetic\_gene\_cluster | 32 | 35.2707006369 | 374 | 9.4e-103 |
| AEZ54377.1 | PieA4 | BGC0000124 | Piericidin\_A1\_biosynthetic\_gene\_cluster | 29 | 46.2181528662 | 374 | 9.4e-103 |
| CAC22144.1 | putative\_type\_I\_polyketide\_synthase | BGC0000038 | Coelimycin\_biosynthetic\_gene\_cluster | 28 | 46.6560509554 | 374 | 9.4e-103 |
| EFY94490.1 | polyketide\_synthase,\_putative | BGC0000337 | Destruxins\_biosynthetic\_gene\_cluster | 27 | 47.5318471338 | 373 | 1.6e-102 |
| AAB66508.1 | tylactone\_synthase\_module\_7 | BGC0000166 | Tylactone\_biosynthetic\_gene\_cluster | 32 | 33.5191082803 | 373 | 1.6e-102 |
| BAH02271.1 | polyketide\_synthase | BGC0000126 | Pladienolide\_biosynthetic\_gene\_cluster | 31 | 38.2961783439 | 372 | 3.6e-102 |
| AAM54076.1 | polyketide\_synthase | BGC0000020 | Ansamitocin\_biosynthetic\_gene\_cluster | 32 | 37.101910828 | 372 | 4.6e-102 |
| AAQ82568.1 | FscD | BGC0000061 | FR-008\_biosynthetic\_gene\_cluster | 29 | 47.1337579618 | 372 | 4.6e-102 |
| BAQ25481.1 | type\_I\_polyketide\_synthase | BGC0001288 | Maklamicin\_biosynthetic\_gene\_cluster | 27 | 48.8455414013 | 372 | 4.6e-102 |
| ABW96540.1 | type\_I\_modular\_polyketide\_synthase | BGC0000159 | Tautomycin\_biosynthetic\_gene\_cluster | 28 | 48.4474522293 | 371 | 6.1e-102 |
| AEZ54375.1 | PieA2 | BGC0000124 | Piericidin\_A1\_biosynthetic\_gene\_cluster | 32 | 34.9522292994 | 371 | 7.9e-102 |
| CAA60459.1 | polyketide\_synthase | BGC0001040 | Rapamycin\_biosynthetic\_gene\_cluster | 29 | 45.6210191083 | 371 | 1e-101 |
| ABC87510.1 | polyketide\_synthase | BGC0001011 | Meridamycin\_biosynthetic\_gene\_cluster | 28 | 48.9649681529 | 371 | 1e-101 |
| ctg1\_orf21 |  | BGC0001013 | Meridamycin\_biosynthetic\_gene\_cluster | 28 | 48.9649681529 | 371 | 1e-101 |
| ACR50785.1 | polyketide\_synthase | BGC0000163 | Tetronasin\_biosynthetic\_gene\_cluster | 28 | 48.6066878981 | 371 | 1e-101 |
| ABV99085.1 | thioester\_reductase\_domain | BGC0001007 | Lymphostin\_biosynthetic\_gene\_cluster | 32 | 36.8232484076 | 370 | 1.4e-101 |
| AAP42857.1 | NanA3 | BGC0000105 | Nanchangmycin\_biosynthetic\_gene\_cluster | 28 | 46.4171974522 | 370 | 1.4e-101 |
| AAO65806.1 | monensin\_polyketide\_synthase\_modules\_11\_and\_12 | BGC0000100 | Monensin\_biosynthetic\_gene\_cluster | 29 | 41.5605095541 | 370 | 1.8e-101 |
| ADB12490.1 | EpoC | BGC0000990 | Epothilone\_biosynthetic\_gene\_cluster | 29 | 49.0843949045 | 370 | 1.8e-101 |
| AAY28227.1 | HbmAIII | BGC0000074 | Herbimycin\_biosynthetic\_gene\_cluster | 28 | 47.8503184713 | 369 | 2.3e-101 |
| ADX66459.1 | ScnS4 | BGC0000108 | Natamycin\_biosynthetic\_gene\_cluster | 28 | 47.7308917197 | 369 | 2.3e-101 |
| CAE46850.1 | Type\_I\_modular\_polyketide\_synthase | BGC0000103 | Mycolactone\_biosynthetic\_gene\_cluster | 28 | 45.3423566879 | 369 | 3e-101 |
| ABC84456.1 | NigAI | BGC0000114 | Nigericin\_biosynthetic\_gene\_cluster | 27 | 47.8901273885 | 369 | 3.9e-101 |
| AAF00958.1 | McyE | BGC0001017 | Microcystin\_biosynthetic\_gene\_cluster | 30 | 38.0573248408 | 369 | 3.9e-101 |
| AFU82617.1 | polyketide\_synthase | BGC0000998 | Guadinomine\_biosynthetic\_gene\_cluster | 31 | 37.5796178344 | 369 | 3.9e-101 |
| ADC79637.1 | TamAI | BGC0001052 | Tirandamycin\_biosynthetic\_gene\_cluster | 31 | 37.3407643312 | 368 | 5.1e-101 |
| BAG84248.1 | putative\_polyketide\_synthase | BGC0000257 | Prodigiosin\_biosynthetic\_gene\_cluster | 33 | 37.3407643312 | 368 | 5.1e-101 |
| ABJ97439.1 | MerC | BGC0001012 | Meridamycin\_biosynthetic\_gene\_cluster | 31 | 38.1767515924 | 368 | 5.1e-101 |
| ADH04658.1 | TugB | BGC0001342 | Thuggacin\_biosynthetic\_gene\_cluster | 32 | 34.7531847134 | 368 | 6.7e-101 |
| AEP40940.1 | polyketide\_synthase\_type\_I | BGC0000021 | Apoptolidin\_biosynthetic\_gene\_cluster | 31 | 37.5796178344 | 368 | 6.7e-101 |
| AMB48442.1 | polyketide\_synthase | BGC0001357 | Carbamidocyclophane\_biosynthetic\_gene\_cluster | 30 | 38.097133758 | 368 | 6.7e-101 |
| CBA11584.1 | polyketide\_synthase\_type\_I | BGC0001046 | Streptolydigin\_biosynthetic\_gene\_cluster | 32 | 38.2961783439 | 368 | 8.8e-101 |
| AAQ90173.1 | polyketide\_synthase\_type\_I | BGC0000128 | Pyoluteorin\_biosynthetic\_gene\_cluster | 31 | 38.097133758 | 368 | 8.8e-101 |
| ADB23403.1 | polyketide\_synthase\_type\_I | BGC0001062 | TLN-05220\_biosynthetic\_gene\_cluster | 28 | 51.6719745223 | 367 | 1.1e-100 |
| AEU17897.1 | putative\_type\_I\_PKS | BGC0001072 | Simocyclinone\_biosynthetic\_gene\_cluster | 29 | 49.0445859873 | 367 | 1.1e-100 |
| AAC68815.1 | FK506\_polyketide\_synthase | BGC0000353 | FK506\_biosynthetic\_gene\_cluster | 29 | 45.8200636943 | 367 | 1.1e-100 |
| CAC20920.1 | PimS3\_protein | BGC0000125 | Pimaricin\_biosynthetic\_gene\_cluster | 29 | 47.4920382166 | 367 | 1.1e-100 |
| ACB46194.1 | polyketide\_synthase | BGC0000989 | Epothilone\_biosynthetic\_gene\_cluster | 29 | 47.0939490446 | 367 | 1.1e-100 |
| AAF62882.1 | EpoC | BGC0000991 | Epothilone\_biosynthetic\_gene\_cluster | 29 | 49.1640127389 | 367 | 1.1e-100 |
| AAU93805.2 | polyketide\_synthase\_modules\_5\_and\_6 | BGC0000054 | Erythromycin\_biosynthetic\_gene\_cluster | 32 | 36.5445859873 | 367 | 1.1e-100 |
| ADM46357.1 | polyketide\_synthase | BGC0000106 | Naphthomycin\_biosynthetic\_gene\_cluster | 29 | 46.2977707006 | 367 | 1.5e-100 |
| BAF85839.1 | modular\_polyketide\_synthase | BGC0000109 | Nemadectin\_biosynthetic\_gene\_cluster | 33 | 33.8375796178 | 366 | 2e-100 |
| AAF26920.1 | polyketide\_synthase | BGC0000988 | Epothilone\_biosynthetic\_gene\_cluster | 29 | 46.9347133758 | 366 | 2e-100 |
| ABJ97438.1 | MerB | BGC0001012 | Meridamycin\_biosynthetic\_gene\_cluster | 31 | 37.1417197452 | 366 | 2e-100 |
| CAD19085.1 | StiA\_protein | BGC0000153 | Stigmatellin\_biosynthetic\_gene\_cluster | 30 | 37.2611464968 | 366 | 2e-100 |
| WP\_063764077.1 | hypothetical\_protein | BGC0001348 | JBIR-100\_biosynthetic\_gene\_cluster | 33 | 34.9920382166 | 366 | 2.5e-100 |
| AGZ15473.1 | putative\_type\_I\_polyketide\_synthase | BGC0001036 | Polyoxypeptin\_biosynthetic\_gene\_cluster | 28 | 55.5334394904 | 366 | 2.5e-100 |
| AFV96142.1 | polyketide\_synthase | BGC0001064 | Cylindrocyclophane\_biosynthetic\_gene\_cluster | 30 | 38.7340764331 | 366 | 3.3e-100 |
| AEK75503.1 | type\_1\_polyketide\_synthase | BGC0000001 | Abyssomicin\_biosynthetic\_gene\_cluster | 32 | 35.3105095541 | 366 | 3.3e-100 |
| BAQ21939.1 | putative\_type\_I\_polyketide\_synthase | BGC0001204 | Versipelostatin\_biosynthetic\_gene\_cluster | 33 | 35.3503184713 | 365 | 4.3e-100 |
| ALA09371.1 | type\_I\_modular\_PKS | BGC0001303 | Lobosamide\_biosynthetic\_gene\_cluster | 30 | 47.1337579618 | 365 | 4.3e-100 |
| ADB12493.1 | EpoF | BGC0000990 | Epothilone\_biosynthetic\_gene\_cluster | 29 | 46.377388535 | 365 | 5.7e-100 |
| CAA60462.1 | polyketide\_synthase | BGC0001040 | Rapamycin\_biosynthetic\_gene\_cluster | 29 | 45.6608280255 | 364 | 7.4e-100 |
| AAP42856.1 | NanA2 | BGC0000105 | Nanchangmycin\_biosynthetic\_gene\_cluster | 31 | 33.8375796178 | 364 | 9.7e-100 |
| CBA11582.1 | polyketide\_synthase\_type\_I | BGC0001046 | Streptolydigin\_biosynthetic\_gene\_cluster | 32 | 34.9522292994 | 364 | 9.7e-100 |
| AGZ15475.1 | putative\_type\_1\_modular\_polyketide\_synthase | BGC0001036 | Polyoxypeptin\_biosynthetic\_gene\_cluster | 30 | 38.3359872611 | 363 | 1.7e-99 |
| ABB88521.1 | polyketide\_synthase\_type\_I | BGC0000050 | E-837\_biosynthetic\_gene\_cluster | 29 | 46.6162420382 | 363 | 2.2e-99 |
| ADC79617.1 | BafAII | BGC0000028 | Bafilomycin\_biosynthetic\_gene\_cluster | 32 | 35.2707006369 | 363 | 2.8e-99 |
| ABY21540.1 | AngAIII | BGC0000018 | Angolamycin\_biosynthetic\_gene\_cluster | 30 | 41.4410828025 | 363 | 2.8e-99 |
| ABX60162.1 | polyketide\_synthase | BGC0000978 | Cylindrospermopsin\_biosynthetic\_gene\_cluster | 28 | 47.0939490446 | 363 | 2.8e-99 |
| AAK57186.1 | MxaB2 | BGC0001022 | Myxalamid\_biosynthetic\_gene\_cluster | 32 | 36.5843949045 | 363 | 2.8e-99 |
| ACY06288.1 | type\_I\_polyketide\_synthase | BGC0001042 | Sanglifehrin\_A\_biosynthetic\_gene\_cluster | 32 | 36.1863057325 | 362 | 3.7e-99 |
| ABC84461.1 | NigAVI | BGC0000114 | Nigericin\_biosynthetic\_gene\_cluster | 30 | 38.5350318471 | 362 | 3.7e-99 |
| BAO66541.1 | type\_I\_polyketide\_synthase | BGC0000042 | Cremimycin\_biosynthetic\_gene\_cluster | 28 | 47.8105095541 | 362 | 3.7e-99 |
| BAT51067.1 | type\_I\_polyketide\_synthase | BGC0001296 | Streptazone\_E\_biosynthetic\_gene\_cluster | 29 | 42.0382165605 | 362 | 4.8e-99 |
| AAF71766.1 | NysI | BGC0000115 | Nystatin\_biosynthetic\_gene\_cluster | 33 | 35.6289808917 | 362 | 4.8e-99 |
| ADF88280.1 | polyketide\_synthase | BGC0000981 | Cylindrospermopsin\_biosynthetic\_gene\_cluster | 28 | 47.0939490446 | 361 | 6.3e-99 |
| ABK32263.1 | AmbH | BGC0000014 | Ambruticin\_biosynthetic\_gene\_cluster | 30 | 38.6544585987 | 361 | 6.3e-99 |
| CAD19093.1 | StiJ\_protein | BGC0000153 | Stigmatellin\_biosynthetic\_gene\_cluster | 31 | 38.1369426752 | 361 | 6.3e-99 |
| AFL48528.1 | laidlomycin\_polyketide\_synthase\_(module\_7\_and\_module\_8) | BGC0000084 | Laidlomycin\_biosynthetic\_gene\_cluster | 31 | 36.9426751592 | 361 | 8.2e-99 |
| AAC46026.1 | polyketide\_synthase\_modules\_4\_and\_5 | BGC0000113 | Niddamycin\_biosynthetic\_gene\_cluster | 30 | 37.2213375796 | 361 | 1.1e-98 |
| BAE93728.1 | type\_I\_polyketide\_synthase | BGC0000164 | Tetronomycin\_biosynthetic\_gene\_cluster | 31 | 36.0270700637 | 361 | 1.1e-98 |
| BAP34739.1 | type\_I\_polyketide\_synthase | BGC0000078 | Incednine\_biosynthetic\_gene\_cluster | 28 | 48.8455414013 | 361 | 1.1e-98 |
| ABV83223.1 | CppK | BGC0000116 | Nystatin-like\_Pseudonocardia\_polyene\_biosynthetic\_gene\_cluster | 29 | 44.7850318471 | 360 | 1.4e-98 |
| AFV30249.1 | polyketide\_synthase | BGC0000075 | Hygrocin\_biosynthetic\_gene\_cluster | 28 | 46.7356687898 | 360 | 1.4e-98 |
| AAO65796.1 | monensin\_polyketide\_synthase\_loading\_module\_and\_module\_1 | BGC0000100 | Monensin\_biosynthetic\_gene\_cluster | 28 | 46.6958598726 | 360 | 1.4e-98 |
| AGY30676.1 | Ann4 | BGC0001298 | 4-Z-annimycin\_biosynthetic\_gene\_cluster | 28 | 46.974522293 | 360 | 1.8e-98 |
| BAA20102.2 | 6-methylsalicylic\_acid\_synthase | BGC0001276 | 6-methylsalicyclic\_acid\_biosynthetic\_gene\_cluster | 28 | 44.148089172 | 360 | 1.8e-98 |
| ctg1\_orf8 |  | BGC0000053 | Elaiophylin\_biosynthetic\_gene\_cluster | 31 | 36.2261146497 | 359 | 2.4e-98 |
| ADX66470.1 | ScnS0 | BGC0000108 | Natamycin\_biosynthetic\_gene\_cluster | 32 | 36.9426751592 | 359 | 3.1e-98 |
| ACR50774.1 | polyketide\_synthase | BGC0000163 | Tetronasin\_biosynthetic\_gene\_cluster | 28 | 47.9299363057 | 359 | 4.1e-98 |
| BAR73017.1 | putative\_PKS\_(KS-AT-KR-ACP-KS-AT-DH-KR-ACP) | BGC0001194 | Hitachimycin\_biosynthetic\_gene\_cluster | 29 | 46.2181528662 | 358 | 5.3e-98 |
| BAC76493.1 | lankamycin\_synthase\_LkmAI | BGC0000085 | Lankamycin\_biosynthetic\_gene\_cluster | 31 | 35.9076433121 | 358 | 5.3e-98 |
| BAB69193.1 |  | BGC0000117 | Oligomycin\_biosynthetic\_gene\_cluster | 33 | 35.1910828025 | 358 | 5.3e-98 |
| ctg1\_orf9 |  | BGC0000053 | Elaiophylin\_biosynthetic\_gene\_cluster | 30 | 36.9824840764 | 358 | 6.9e-98 |
| AAP42858.1 | NanA4 | BGC0000105 | Nanchangmycin\_biosynthetic\_gene\_cluster | 28 | 46.8152866242 | 358 | 6.9e-98 |
| ACO94456.1 | polyketide\_synthase\_type\_I | BGC0000029 | BE-14106\_biosynthetic\_gene\_cluster | 30 | 41.7993630573 | 358 | 9.1e-98 |
| AEU17898.1 | putative\_type\_I\_PKS | BGC0001072 | Simocyclinone\_biosynthetic\_gene\_cluster | 30 | 38.1369426752 | 357 | 1.2e-97 |
| ctg1\_orf27 |  | BGC0000096 | Midecamycin\_biosynthetic\_gene\_cluster | 32 | 34.9124203822 | 356 | 2e-97 |
| AAO65798.1 | monensin\_polyketide\_synthase\_modules\_3\_and\_4 | BGC0000100 | Monensin\_biosynthetic\_gene\_cluster | 30 | 42.1576433121 | 356 | 2e-97 |
| CUW01174.1 | Type\_I\_polyketide\_synthase | BGC0001359 | PM100117\_/\_PM100118\_biosynthetic\_gene\_cluster | 30 | 38.4156050955 | 356 | 2e-97 |
| ADC45534.1 | modular\_polyketide\_synthase | BGC0000093 | Meilingmycin\_biosynthetic\_gene\_cluster | 31 | 33.9171974522 | 356 | 2.6e-97 |
| CAQ18833.1 | polyketide\_synthase | BGC0000954 | Ajudazol\_biosynthetic\_gene\_cluster | 30 | 38.2563694268 | 356 | 2.6e-97 |
| AEP40934.1 | polyketide\_synthase\_type\_I | BGC0000021 | Apoptolidin\_biosynthetic\_gene\_cluster | 28 | 48.0493630573 | 356 | 2.6e-97 |
| AHA38203.1 | GphJ | BGC0000069 | Gephyronic\_acid\_biosynthetic\_gene\_cluster | 31 | 38.1369426752 | 355 | 5.9e-97 |
| CAI94713.1 | putative\_polyketide\_synthase | BGC0000141 | Rubradirin\_biosynthetic\_gene\_cluster | 29 | 43.7101910828 | 355 | 5.9e-97 |
| AMB48441.1 | polyketide\_synthase | BGC0001357 | Carbamidocyclophane\_biosynthetic\_gene\_cluster | 29 | 38.2961783439 | 354 | 7.7e-97 |
| AEK75504.1 | type\_1\_polyketide\_synthase | BGC0000001 | Abyssomicin\_biosynthetic\_gene\_cluster | 32 | 36.1066878981 | 354 | 1.3e-96 |
| AEU17899.1 | putative\_type\_I\_PKS | BGC0001072 | Simocyclinone\_biosynthetic\_gene\_cluster | 32 | 35.071656051 | 353 | 1.7e-96 |
| AFL48527.1 | laidlomycin\_polyketide\_synthase\_(module\_3\_and\_module\_4) | BGC0000084 | Laidlomycin\_biosynthetic\_gene\_cluster | 32 | 34.076433121 | 353 | 1.7e-96 |
| AKD43761.1 | HerD | BGC0001349 | Heronamide\_biosynthetic\_gene\_cluster | 29 | 46.377388535 | 353 | 1.7e-96 |
| ABB88536.1 | polyketide\_synthase\_type\_I | BGC0000049\_c2 | E-492\_/\_E-975\_biosynthetic\_gene\_cluster | 30 | 37.6592356688 | 353 | 2.2e-96 |
| ABM21569.1 | CrpA | BGC0000975 | Cryptophycin\_biosynthetic\_gene\_cluster | 28 | 49.5222929936 | 353 | 2.2e-96 |
| ADC79639.1 | TamAIII | BGC0001052 | Tirandamycin\_biosynthetic\_gene\_cluster | 31 | 36.4251592357 | 353 | 2.2e-96 |
| AEK75502.1 | type\_1\_polyketide\_synthase | BGC0000001 | Abyssomicin\_biosynthetic\_gene\_cluster | 31 | 35.9872611465 | 352 | 2.9e-96 |
| AAK73513.1 | AmphB | BGC0000015 | Amphotericin\_biosynthetic\_gene\_cluster | 31 | 37.1815286624 | 352 | 3.8e-96 |
| AAQ84147.1 | Plm7 | BGC0000123 | Phoslactomycin\_B\_biosynthetic\_gene\_cluster | 32 | 34.3949044586 | 352 | 3.8e-96 |
| CBW54671.1 | polyketide\_synthase/non\_ribosomal\_peptide\_synthetase | BGC0000971 | Cinnabaramide\_biosynthetic\_gene\_cluster | 32 | 36.7436305732 | 352 | 3.8e-96 |
| CAM00064.1 | EryAII\_Erythromycin\_polyketide\_synthase\_modules\_3\_and\_4 | BGC0000055 | Erythromycin\_biosynthetic\_gene\_cluster | 28 | 45.8996815287 | 352 | 5e-96 |
| AFL48533.1 | laidlomycin\_polyketide\_synthase\_(module\_10) | BGC0000084 | Laidlomycin\_biosynthetic\_gene\_cluster | 31 | 36.2261146497 | 352 | 5e-96 |
| AAG23265.1 | polyketide\_synthase\_extender\_module\_2 | BGC0000148 | Spinosad\_biosynthetic\_gene\_cluster | 27 | 51.8710191083 | 352 | 5e-96 |
| AHH99922.1 | PKS\_I | BGC0000002 | Aculeximycin\_biosynthetic\_gene\_cluster | 32 | 34.5541401274 | 352 | 5e-96 |
| AAW03327.1 | CtaD | BGC0000982 | Cystothiazole\_A\_biosynthetic\_gene\_cluster | 27 | 48.3280254777 | 351 | 8.5e-96 |
| CAD15508.1 | probable\_polyketide\_synthase\_protein | BGC0001014 | Micacocidin\_biosynthetic\_gene\_cluster | 31 | 36.7834394904 | 351 | 8.5e-96 |
| ctg1\_orf10 |  | BGC0000053 | Elaiophylin\_biosynthetic\_gene\_cluster | 29 | 38.3757961783 | 351 | 1.1e-95 |
| AAP42867.1 | NanA7 | BGC0000105 | Nanchangmycin\_biosynthetic\_gene\_cluster | 30 | 38.1767515924 | 351 | 1.1e-95 |
| WP\_043476519.1 | polyketide\_synthase | BGC0001232 | Leucanicidin\_biosynthetic\_gene\_cluster | 31 | 35.1114649682 | 350 | 1.4e-95 |
| BAF85837.1 | modular\_polyketide\_synthase | BGC0000109 | Nemadectin\_biosynthetic\_gene\_cluster | 29 | 48.4872611465 | 350 | 1.4e-95 |
| AAF86393.1 | FkbB | BGC0000994 | FK520\_biosynthetic\_gene\_cluster | 28 | 47.571656051 | 350 | 1.4e-95 |
| ACB37741.1 | putative\_type\_I\_polyketide\_synthase | BGC0000162 | Tetrocarcin\_A\_biosynthetic\_gene\_cluster | 28 | 46.974522293 | 350 | 1.9e-95 |
| AGZ15472.1 | putative\_modular\_polyketide\_synthase | BGC0001036 | Polyoxypeptin\_biosynthetic\_gene\_cluster | 31 | 38.097133758 | 350 | 1.9e-95 |
| AIG62146.1 | 6-methylsalicylic\_acid\_synthase | BGC0000120 | Patulin\_biosynthetic\_gene\_cluster | 29 | 42.5557324841 | 349 | 2.5e-95 |
| ACN69988.1 | polyketide\_synthase | BGC0000079 | Indanomycin\_biosynthetic\_gene\_cluster | 30 | 40.4458598726 | 349 | 2.5e-95 |
| ADH04657.1 | TugA | BGC0001342 | Thuggacin\_biosynthetic\_gene\_cluster | 32 | 37.0621019108 | 349 | 4.2e-95 |
| AAC46024.1 | polyketide\_synthase\_modules\_1\_and\_2 | BGC0000113 | Niddamycin\_biosynthetic\_gene\_cluster | 31 | 35.1512738854 | 348 | 5.5e-95 |
| BAO66539.1 | type\_I\_polyketide\_synthase | BGC0000042 | Cremimycin\_biosynthetic\_gene\_cluster | 31 | 36.9824840764 | 348 | 5.5e-95 |
| CAO85897.1 | modular\_polyketide\_synthase\_NorB | BGC0000110 | Neoaureothin\_biosynthetic\_gene\_cluster | 28 | 47.0541401274 | 348 | 7.2e-95 |
| ABC84459.1 | NigAIV | BGC0000114 | Nigericin\_biosynthetic\_gene\_cluster | 28 | 46.576433121 | 347 | 9.4e-95 |
| AHB82065.1 | polyketide\_synthase | BGC0001231 | Microsclerodermins\_biosynthetic\_gene\_cluster | 36 | 27.6671974522 | 347 | 1.2e-94 |
| CAQ34929.1 | putative\_polyketide\_synthase | BGC0000986 | Dkxanthene\_biosynthetic\_gene\_cluster | 31 | 33.5987261146 | 347 | 1.6e-94 |
| AAQ82564.1 | FscC | BGC0000061 | FR-008\_biosynthetic\_gene\_cluster | 27 | 47.6512738854 | 346 | 2.1e-94 |
| AAP42860.1 | NanA6 | BGC0000105 | Nanchangmycin\_biosynthetic\_gene\_cluster | 31 | 37.101910828 | 346 | 2.1e-94 |
| ABG02264.1 | SalB | BGC0000143 | Salinomycin\_biosynthetic\_gene\_cluster | 31 | 37.4601910828 | 346 | 2.7e-94 |
| CAC20931.1 | PimS1\_protein | BGC0000125 | Pimaricin\_biosynthetic\_gene\_cluster | 32 | 33.3996815287 | 346 | 2.7e-94 |
| AAZ77697.1 | ChlA4 | BGC0000036 | Chlorothricin\_biosynthetic\_gene\_cluster | 27 | 47.7707006369 | 346 | 2.7e-94 |
| CAQ18838.1 | polyketide\_synthase | BGC0000954 | Ajudazol\_biosynthetic\_gene\_cluster | 31 | 37.0222929936 | 346 | 2.7e-94 |
| AHH99921.1 | PKS\_I | BGC0000002 | Aculeximycin\_biosynthetic\_gene\_cluster | 28 | 52.0302547771 | 346 | 2.7e-94 |
| BAB69192.1 | modular\_polyketide\_synthase | BGC0000117 | Oligomycin\_biosynthetic\_gene\_cluster | 33 | 32.4442675159 | 346 | 3.6e-94 |
| ctg1\_orf7 |  | BGC0000053 | Elaiophylin\_biosynthetic\_gene\_cluster | 30 | 37.1815286624 | 346 | 3.6e-94 |
| ACR50782.1 | polyketide\_synthase | BGC0000163 | Tetronasin\_biosynthetic\_gene\_cluster | 32 | 33.7579617834 | 345 | 4.7e-94 |
| EHA22196.1 | polyketide\_synthase | BGC0000170 | Yanuthone\_D\_biosynthetic\_gene\_cluster | 31 | 36.8232484076 | 345 | 4.7e-94 |
| AAP33839.2 | ochratoxin\_A\_polyketide\_synthase | BGC0001030 | Ochratoxin\_A\_biosynthetic\_gene\_cluster | 32 | 30.9713375796 | 345 | 4.7e-94 |
| ACO94484.1 | polyketide\_synthase\_type\_I | BGC0000097 | ML-449\_biosynthetic\_gene\_cluster | 29 | 41.3614649682 | 344 | 7.9e-94 |
| AAG13917.1 | megalomicin\_6-deoxyerythronolide\_B\_synthase\_1 | BGC0000092 | Megalomicin\_biosynthetic\_gene\_cluster | 30 | 38.1767515924 | 344 | 7.9e-94 |
| AAF19812.1 | MtaD | BGC0001024 | Myxothiazol\_biosynthetic\_gene\_cluster | 28 | 48.8455414013 | 344 | 1e-93 |
| ABB88533.1 | polyketide\_synthase\_type\_I | BGC0000049 | E-492\_/\_E-975\_biosynthetic\_gene\_cluster | 32 | 33.4394904459 | 344 | 1.4e-93 |
| AAO62584.1 | polyketide\_synthase\_type\_1 | BGC0001016 | Microcystin\_biosynthetic\_gene\_cluster | 27 | 48.7261146497 | 343 | 1.8e-93 |
| AHA38199.1 | GphF | BGC0000069 | Gephyronic\_acid\_biosynthetic\_gene\_cluster | 28 | 41.5605095541 | 342 | 3.9e-93 |
| CAD89775.1 | MelD\_protein | BGC0001010 | Melithiazol\_biosynthetic\_gene\_cluster | 27 | 46.5366242038 | 342 | 3.9e-93 |
| ADX66461.1 | ScnS2 | BGC0000108 | Natamycin\_biosynthetic\_gene\_cluster | 31 | 36.7038216561 | 342 | 3.9e-93 |
| BAD08359.1 | polyketide\_synthase\_modules\_5-6 | BGC0000167 | Vicenistatin\_biosynthetic\_gene\_cluster | 31 | 37.4601910828 | 341 | 6.7e-93 |
| BAF85838.1 | modular\_polyketide\_synthase | BGC0000109 | Nemadectin\_biosynthetic\_gene\_cluster | 31 | 36.7834394904 | 341 | 8.8e-93 |
| EAU32819.1 | 6-methylsalicylic\_acid\_synthase | BGC0000160 | Terreic\_acid\_biosynthetic\_gene\_cluster | 29 | 40.6847133758 | 340 | 1.5e-92 |
| AGI99496.1 | Type\_I\_polyketide\_synthase | BGC0001004 | Lobophorin\_biosynthetic\_gene\_cluster | 30 | 41.6401273885 | 340 | 2e-92 |
| CBZ41586.1 | putative\_modular\_polyketide\_synthase | BGC0000151 | Stambomycin\_biosynthetic\_gene\_cluster | 31 | 33.3200636943 | 340 | 2e-92 |
| AHA12079.1 | polyketide\_synthase\_type\_1 | BGC0001172 | Chlorizidine\_A\_biosynthetic\_gene\_cluster | 31 | 37.5796178344 | 339 | 2.6e-92 |
| BAQ25482.1 | type\_I\_polyketide\_synthase | BGC0001288 | Maklamicin\_biosynthetic\_gene\_cluster | 28 | 47.9299363057 | 339 | 4.4e-92 |
| AAZ94386.1 | modular\_polyketide\_synthase | BGC0000040 | Concanamycin\_A\_biosynthetic\_gene\_cluster | 31 | 36.8630573248 | 338 | 7.4e-92 |
| AAQ84144.1 | Plm4 | BGC0000123 | Phoslactomycin\_B\_biosynthetic\_gene\_cluster | 30 | 35.9474522293 | 338 | 7.4e-92 |
| ctg1\_orf20 |  | BGC0001013 | Meridamycin\_biosynthetic\_gene\_cluster | 31 | 35.2308917197 | 337 | 9.7e-92 |
| ABC87509.1 | polyketide\_synthase | BGC0001011 | Meridamycin\_biosynthetic\_gene\_cluster | 31 | 35.2308917197 | 337 | 9.7e-92 |
| AEZ53947.1 | polyketide\_synthase | BGC0000144 | Salinomycin\_biosynthetic\_gene\_cluster | 31 | 34.872611465 | 337 | 1.3e-91 |
| AAO65807.1 | monensin\_polyketide\_synthase\_module\_10 | BGC0000100 | Monensin\_biosynthetic\_gene\_cluster | 29 | 38.6146496815 | 336 | 2.8e-91 |
| CAQ52624.1 | type\_I\_polyketide\_synthase,\_modules\_7-8 | BGC0001066 | Kendomycin\_biosynthetic\_gene\_cluster | 27 | 48.4872611465 | 336 | 3.7e-91 |
| CCE67070.1 | polyketide\_synthase | BGC0001242 | Fusarubin\_biosynthetic\_gene\_cluster | 30 | 38.097133758 | 335 | 4.8e-91 |
| AAF86396.1 | FkbA | BGC0000994 | FK520\_biosynthetic\_gene\_cluster | 31 | 39.0127388535 | 335 | 4.8e-91 |
| AAY89049.1 | polyketide\_synthase | BGC0001069 | Chivosazole\_biosynthetic\_gene\_cluster | 31 | 37.5796178344 | 335 | 6.3e-91 |
| BAE93722.1 | type\_I\_polyketide\_synthase | BGC0000164 | Tetronomycin\_biosynthetic\_gene\_cluster | 32 | 35.6289808917 | 334 | 8.2e-91 |
| CAE45668.1 | borrelidin\_polyketide\_synthase,\_type\_I | BGC0000031 | Borrelidin\_biosynthetic\_gene\_cluster | 31 | 35.0318471338 | 334 | 8.2e-91 |
| AEC13079.1 | fosA | BGC0000060 | Fostriecin\_biosynthetic\_gene\_cluster | 33 | 29.6576433121 | 333 | 1.8e-90 |
| ABJ97437.1 | MerA | BGC0001012 | Meridamycin\_biosynthetic\_gene\_cluster | 32 | 35.9076433121 | 332 | 3.1e-90 |
| CAO98848.1 | polyketide\_synthase\_AufE | BGC0000023 | Aurafuron\_biosynthetic\_gene\_cluster | 34 | 27.6671974522 | 332 | 3.1e-90 |
| AGC09484.1 | LobS1 | BGC0001183 | Lobophorin\_biosynthetic\_gene\_cluster | 29 | 38.097133758 | 332 | 4.1e-90 |
| AJW65407.1 | type\_I\_modular\_polyketide\_synthase | BGC0001195 | Nocardiopsin\_biosynthetic\_gene\_cluster | 30 | 37.699044586 | 331 | 7e-90 |
| AGI99497.1 | type\_I\_polyketide\_synthase | BGC0001004 | Lobophorin\_biosynthetic\_gene\_cluster | 29 | 38.097133758 | 331 | 9.1e-90 |
| AAP85336.1 | type\_I\_PKS | BGC0000233 | Hedamycin\_biosynthetic\_gene\_cluster | 30 | 37.0222929936 | 331 | 9.1e-90 |
| CAQ18839.1 | hybrid\_polyketide\_synthase/nonribosomal\_polypetide\_synthetase | BGC0000954 | Ajudazol\_biosynthetic\_gene\_cluster | 30 | 39.0127388535 | 330 | 2e-89 |
| CCP20051.1 | divM\_protein | BGC0001119 | Divergolide\_biosynthetic\_gene\_cluster | 30 | 35.0318471338 | 330 | 2e-89 |
| CAE02605.1 | polyketide\_synthase\_type\_I | BGC0000024 | Aureothin\_biosynthetic\_gene\_cluster | 31 | 34.2356687898 | 329 | 2.6e-89 |
| EAL84397.1 | polyketide\_synthase,\_putative | BGC0001118 | Endocrocin\_biosynthetic\_gene\_cluster | 29 | 38.1767515924 | 328 | 7.7e-89 |
| AJO72737.1 | Type\_I\_modular\_polyketide\_synthase | BGC0001381 | Brasilinolide\_biosynthetic\_gene\_cluster | 30 | 38.4156050955 | 327 | 1e-88 |
| AHA38200.1 | GphG | BGC0000069 | Gephyronic\_acid\_biosynthetic\_gene\_cluster | 32 | 34.7531847134 | 326 | 2.9e-88 |
| AAQ82567.1 | FscE | BGC0000061 | FR-008\_biosynthetic\_gene\_cluster | 30 | 41.4808917197 | 325 | 5e-88 |
| AJW65408.1 | type\_I\_modular\_polyketide\_synthase | BGC0001195 | Nocardiopsin\_biosynthetic\_gene\_cluster | 30 | 39.0525477707 | 325 | 6.5e-88 |
| ABB88535.1 | polyketide\_synthase\_type\_I | BGC0000049\_c2 | E-492\_/\_E-975\_biosynthetic\_gene\_cluster | 28 | 49.1640127389 | 324 | 8.5e-88 |
| ABV83222.1 | CppJ | BGC0000116 | Nystatin-like\_Pseudonocardia\_polyene\_biosynthetic\_gene\_cluster | 28 | 46.5366242038 | 324 | 1.4e-87 |
| AAN32979.1 | BarE | BGC0000962 | Barbamide\_biosynthetic\_gene\_cluster | 28 | 39.5302547771 | 324 | 1.4e-87 |
| CAQ18830.1 | polyketide\_synthase | BGC0000954 | Ajudazol\_biosynthetic\_gene\_cluster | 33 | 29.0207006369 | 323 | 1.9e-87 |
| ACB46487.1 | polyketide\_synthase | BGC0000082 | Kijanimicin\_biosynthetic\_gene\_cluster | 29 | 41.7197452229 | 322 | 3.2e-87 |
| AAZ77698.1 | ChlA5 | BGC0000036 | Chlorothricin\_biosynthetic\_gene\_cluster | 31 | 35.5891719745 | 322 | 5.5e-87 |
| AHE80995.1 | PieA5 | BGC0001169 | Piericidin\_A1\_biosynthetic\_gene\_cluster | 28 | 47.173566879 | 321 | 7.2e-87 |
| AJO72743.1 | Type\_I\_modular\_polyketide\_synthase | BGC0001381 | Brasilinolide\_biosynthetic\_gene\_cluster | 31 | 33.5589171975 | 321 | 1.2e-86 |
| AAO65800.1 | monensin\_polyketide\_synthase\_modules\_7\_and\_8 | BGC0000100 | Monensin\_biosynthetic\_gene\_cluster | 27 | 46.974522293 | 319 | 3.6e-86 |
| ABY21542.1 | AngAV | BGC0000018 | Angolamycin\_biosynthetic\_gene\_cluster | 31 | 34.9920382166 | 317 | 1e-85 |
| CAQ18832.1 | polyketide\_synthase | BGC0000954 | Ajudazol\_biosynthetic\_gene\_cluster | 33 | 28.7818471338 | 317 | 1.8e-85 |
| AAS90093.1 | PksA | BGC0000006 | Aflatoxin\_biosynthetic\_gene\_cluster | 30 | 38.6544585987 | 316 | 3e-85 |
| CBF74114.1 | Conidial\_yellow\_pigment\_biosynthesis\_polyketide\_synthase\_(PKS)(EC\_2.3.1.-)\_[Source:UniProtKB/Swiss-Prot;Acc:Q03149] | BGC0000107 | Naphthopyrone\_biosynthetic\_gene\_cluster | 29 | 38.3359872611 | 316 | 3.9e-85 |
| AAD03048.1 | type\_I\_polyketide\_synthase | BGC0000041 | Coronafacic\_acid\_biosynthetic\_gene\_cluster | 31 | 36.6640127389 | 315 | 6.7e-85 |
| ABB88519.1 | polyketide\_synthase\_type\_I | BGC0000050 | E-837\_biosynthetic\_gene\_cluster | 32 | 37.6592356688 | 314 | 1.5e-84 |
| BAE71314.1 | polyketide\_synthase | BGC0000004 | Aflatoxin\_biosynthetic\_gene\_cluster | 29 | 38.6544585987 | 313 | 2.6e-84 |
| AHV78253.1 | ResS2 | BGC0001246 | Trans-resorcylide\_biosynthetic\_gene\_cluster | 29 | 38.8535031847 | 313 | 2.6e-84 |
| AAS90022.1 | PksA | BGC0000008 | Aflatoxin\_biosynthetic\_gene\_cluster | 30 | 38.8136942675 | 313 | 2.6e-84 |
| ABV83221.1 | CppI | BGC0000116 | Nystatin-like\_Pseudonocardia\_polyene\_biosynthetic\_gene\_cluster | 29 | 35.6289808917 | 312 | 3.3e-84 |
| AAS89999.1 | PksA | BGC0000007 | Aflatoxin\_biosynthetic\_gene\_cluster | 30 | 38.6544585987 | 312 | 4.4e-84 |
| ACH72912.1 | AflC | BGC0000011 | Aflatoxin/sterigmatocystin\_biosynthetic\_gene\_cluster | 29 | 39.5302547771 | 311 | 9.7e-84 |
| AAF71776.1 | NysC | BGC0000115 | Nystatin\_biosynthetic\_gene\_cluster | 30 | 37.0222929936 | 311 | 1.3e-83 |
| AAO62585.1 | peptide\_sythetase\_polyketide\_synthase\_fusion\_protein | BGC0001016 | Microcystin\_biosynthetic\_gene\_cluster | 30 | 37.699044586 | 310 | 2.2e-83 |
| CAN89636.1 | putative\_polyketide\_synthase | BGC0001070 | Kirromycin\_biosynthetic\_gene\_cluster | 26 | 47.7308917197 | 309 | 3.7e-83 |
| AHV78247.1 | LasS2 | BGC0001245 | Lasiodiplodin\_biosynthetic\_gene\_cluster | 30 | 37.8184713376 | 309 | 3.7e-83 |
| EAL89339.1 | polyketide\_synthase,\_putative | BGC0001403 | Trypacidin\_biosynthetic\_gene\_cluster | 28 | 38.5748407643 | 308 | 6.3e-83 |
| AAR87760.2 | ZmaK | BGC0001059 | Zwittermycin\_A\_biosynthetic\_gene\_cluster | 29 | 38.0573248408 | 308 | 6.3e-83 |
| CBF90097.1 | hypothetical\_protein\_similar\_to\_polyketide\_synthase\_(Broad) | BGC0001271 | Emericellin\_biosynthetic\_gene\_cluster | 29 | 38.4156050955 | 307 | 1.1e-82 |
| ACD39770.1 | non-reducing\_polyketide\_synthase | BGC0000134 | Radicicol\_biosynthetic\_gene\_cluster | 29 | 38.2563694268 | 307 | 1.4e-82 |
| AIW00670.1 | mellein\_synthase | BGC0001244 | (-)-Mellein\_biosynthetic\_gene\_cluster | 25 | 45.3821656051 | 307 | 1.8e-82 |
| ACA99172.1 | Polyketide\_synthase | BGC0001160 | Nonadecene\_biosynthetic\_gene\_cluster | 29 | 38.4156050955 | 306 | 2.4e-82 |
| AAS90047.1 | PksA | BGC0000009 | Aflatoxin\_biosynthetic\_gene\_cluster | 29 | 38.2961783439 | 306 | 2.4e-82 |
| CAD70195.1 | non-ribosomal\_peptide\_synthetase | BGC0001047 | Syringolin\_A\_biosynthetic\_gene\_cluster | 27 | 46.4570063694 | 306 | 3.1e-82 |
| AGC95321.1 | CurS2 | BGC0000045 | Dehydrocurvularin\_biosynthetic\_gene\_cluster | 29 | 40.0477707006 | 305 | 5.3e-82 |
| AFN27480.1 | pks\_BonA | BGC0000173 | Bongkrekic\_acid\_biosynthetic\_gene\_cluster | 37 | 21.576433121 | 305 | 5.3e-82 |
| CCH26295.1 | putative\_polyketide\_synthase | BGC0001158\_c6 | Bikaverin\_biosynthetic\_gene\_cluster | 29 | 38.1767515924 | 304 | 9.1e-82 |
| AHB82072.1 | non\_ribosomal\_peptide\_synthetase/polyketide\_synthase | BGC0001231 | Microsclerodermins\_biosynthetic\_gene\_cluster | 30 | 36.4649681529 | 304 | 1.6e-81 |
| CCT67991.1 | bikaverin\_cluster-polyketide\_synthase | BGC0000030 | Bikaverin\_biosynthetic\_gene\_cluster | 28 | 37.9378980892 | 301 | 1e-80 |
| AAF15892.2 | NosB | BGC0001028 | Nostopeptolide\_biosynthetic\_gene\_cluster | 29 | 36.4251592357 | 300 | 1.3e-80 |
| ADY00130.1 | polyketide\_synthase | BGC0000104 | Mycophenolic\_acid\_biosynthetic\_gene\_cluster | 27 | 48.3678343949 | 300 | 2.2e-80 |
| AGO59040.1 | PtaA | BGC0000121 | Pestheic\_acid\_biosynthetic\_gene\_cluster | 28 | 38.6146496815 | 299 | 2.9e-80 |
| AEU11005.1 | NpnA | BGC0001029 | Nostophycin\_biosynthetic\_gene\_cluster | 26 | 45.3423566879 | 297 | 1.9e-79 |
| ADF88275.1 | polyketide\_synthase | BGC0000981 | Cylindrospermopsin\_biosynthetic\_gene\_cluster | 28 | 38.4554140127 | 295 | 7.2e-79 |
| AAK73512.1 | AmphA | BGC0000015 | Amphotericin\_biosynthetic\_gene\_cluster | 26 | 48.0891719745 | 295 | 7.2e-79 |
| AAF00957.1 | McyG | BGC0001017 | Microcystin\_biosynthetic\_gene\_cluster | 29 | 39.1719745223 | 293 | 2.7e-78 |
| ADA69241.1 | cis-AT\_polyketide\_synthase | BGC0001071 | Nosperin\_biosynthetic\_gene\_cluster | 28 | 35.7882165605 | 291 | 8e-78 |
| CAP95404.1 |  | BGC0001404 | Sorbicillin\_biosynthetic\_gene\_cluster | 25 | 48.5668789809 | 287 | 1.2e-76 |
| ctg1\_orf521 |  | BGC0001199 | Akaeolide\_biosynthetic\_gene\_cluster | 33 | 28.3439490446 | 287 | 1.2e-76 |
| ACY06287.1 | type\_I\_polyketide\_synthase | BGC0001042 | Sanglifehrin\_A\_biosynthetic\_gene\_cluster | 35 | 20.2627388535 | 287 | 1.5e-76 |
| CAD29793.1 | polyketide\_synthase\_type\_I | BGC0001015 | Microcystin\_biosynthetic\_gene\_cluster | 33 | 27.1098726115 | 287 | 1.5e-76 |
| AAS92537.1 | PKS1 | BGC0001044 | Sirodesmin\_biosynthetic\_gene\_cluster | 29 | 38.1767515924 | 287 | 1.5e-76 |
| AJO72736.1 | Type\_I\_modular\_polyketide\_synthase | BGC0001381 | Brasilinolide\_biosynthetic\_gene\_cluster | 29 | 37.8184713376 | 287 | 1.5e-76 |
| AMH40422.1 | PKS | BGC0001350 | Phormidolide\_biosynthetic\_gene\_cluster | 34 | 21.6958598726 | 284 | 1.3e-75 |
| ABX60153.1 | polyketide\_synthase | BGC0000978 | Cylindrospermopsin\_biosynthetic\_gene\_cluster | 27 | 38.3359872611 | 283 | 2.2e-75 |
| AHD05615.1 | putative\_non-ribosomal\_peptide\_ligase/\_polyketide\_synthase\_hybrid | BGC0001033 | Paenilamicin\_biosynthetic\_gene\_cluster | 28 | 39.0923566879 | 283 | 2.8e-75 |
| CAE45672.1 | Borrelidin\_polyketide\_synthase,\_type\_I | BGC0000031 | Borrelidin\_biosynthetic\_gene\_cluster | 29 | 35.4299363057 | 282 | 3.7e-75 |
| ADI59531.1 | CorI | BGC0001091 | Corallopyronin\_biosynthetic\_gene\_cluster | 36 | 21.3375796178 | 282 | 4.8e-75 |
| AKQ22699.1 | malonyl\_CoA-acyl\_carrier\_protein\_transacylase | BGC0001186 | Misakinolide\_biosynthetic\_gene\_cluster | 33 | 24.601910828 | 282 | 6.3e-75 |
| AFD30954.1 | CrmA | BGC0000966 | Caerulomycin\_A\_biosynthetic\_gene\_cluster | 29 | 37.101910828 | 282 | 6.3e-75 |
| ABS90471.1 | PKS\_type\_I | BGC0001106 | Oxazolomycin\_biosynthetic\_gene\_cluster | 31 | 31.8073248408 | 281 | 8.3e-75 |
| CAE45669.1 | borrelidin\_polyketide\_synthase,\_type\_I | BGC0000031 | Borrelidin\_biosynthetic\_gene\_cluster | 27 | 46.377388535 | 281 | 1.1e-74 |
| AAN59953.1 | polyketide\_synthase\_1 | BGC0001258 | 1,8-dihydroxynaphthalene\_biosynthetic\_gene\_cluster | 26 | 46.5366242038 | 280 | 2.4e-74 |
| AAY32964.1 | DszA | BGC0001093 | Disorazole\_biosynthetic\_gene\_cluster | 32 | 27.5477707006 | 279 | 5.3e-74 |
| CCC55921.1 | non-ribosomal\_peptide\_synthetase/polyketide\_synthase\_hybrid\_protein | BGC0000973 | Collismycin\_A\_biosynthetic\_gene\_cluster | 28 | 37.8582802548 | 277 | 1.2e-73 |
| ACN69992.1 | polyketide\_synthase | BGC0000079 | Indanomycin\_biosynthetic\_gene\_cluster | 30 | 27.7070063694 | 277 | 1.6e-73 |
| CAF05649.1 | TubD\_protein | BGC0001053 | Tubulysin\_biosynthetic\_gene\_cluster | 27 | 39.0127388535 | 277 | 1.6e-73 |
| ABP57747.1 | DepC | BGC0000993 | FK228\_biosynthetic\_gene\_cluster | 34 | 21.9347133758 | 276 | 2.7e-73 |
| AHE80993.1 | PieA3 | BGC0001169 | Piericidin\_A1\_biosynthetic\_gene\_cluster | 30 | 29.9761146497 | 276 | 3.5e-73 |
| AAP42872.1 | NanA9 | BGC0000105 | Nanchangmycin\_biosynthetic\_gene\_cluster | 32 | 27.2292993631 | 276 | 3.5e-73 |
| AAD38786.1 | polyketide\_synthase | BGC0001257 | 1,3,6,8-tetrahydroxynaphthalene\_biosynthetic\_gene\_cluster | 28 | 37.8582802548 | 275 | 5.9e-73 |
| ADF88277.1 | polyketide\_synthase | BGC0000981 | Cylindrospermopsin\_biosynthetic\_gene\_cluster | 34 | 20.7006369427 | 274 | 1e-72 |
| ACY01391.1 | AT-less\_polyketide\_synthase | BGC0000177 | Dorrigocin\_/\_migrastatin\_biosynthetic\_gene\_cluster | 35 | 21.2977707006 | 274 | 1e-72 |
| ACN69991.1 | polyketide\_synthase | BGC0000079 | Indanomycin\_biosynthetic\_gene\_cluster | 32 | 27.7468152866 | 274 | 1.7e-72 |
| AGN11881.1 | TstDEF | BGC0001114 | Thailanstatin\_biosynthetic\_gene\_cluster | 35 | 21.974522293 | 274 | 1.7e-72 |
| ABI93779.1 | GdmPKS | BGC0000068 | Geldanamycin\_biosynthetic\_gene\_cluster | 31 | 31.0907643312 | 273 | 2.2e-72 |
| ABX60163.1 | polyketide\_synthase | BGC0000978 | Cylindrospermopsin\_biosynthetic\_gene\_cluster | 34 | 20.7006369427 | 273 | 2.2e-72 |
| CAN93350.1 | polyketide\_synthase | BGC0000179 | Etnangien\_biosynthetic\_gene\_cluster | 33 | 21.6162420382 | 273 | 2.9e-72 |
| EED53479.1 | polyketide\_synthase,\_putative | BGC0001304 | Aflavarin\_biosynthetic\_gene\_cluster | 28 | 38.2563694268 | 272 | 6.5e-72 |
| AIC32693.1 | FR9DEF | BGC0001113 | Spliceostatin\_/\_FR901464\_biosynthetic\_gene\_cluster | 33 | 23.9251592357 | 271 | 1.1e-71 |
| ADH01487.1 | polyketide\_synthase | BGC0001096 | FR901464\_biosynthetic\_gene\_cluster | 33 | 23.9251592357 | 271 | 1.1e-71 |
| CTQ34883.1 | AtcF;\_polyketide\_synthase,\_modules\_8-10 | BGC0001301 | Anthracimycin\_biosynthetic\_gene\_cluster | 33 | 23.8853503185 | 271 | 1.1e-71 |
| AAQ82565.1 | FscB | BGC0000061 | FR-008\_biosynthetic\_gene\_cluster | 34 | 20.7006369427 | 270 | 1.5e-71 |
| CAQ18828.1 | polyketide\_synthase | BGC0000954 | Ajudazol\_biosynthetic\_gene\_cluster | 36 | 20.3423566879 | 270 | 1.9e-71 |
| CCP20050.1 | divL3\_protein | BGC0001119 | Divergolide\_biosynthetic\_gene\_cluster | 30 | 28.1449044586 | 268 | 7.2e-71 |
| CAL58681.1 | polyketide\_synthase | BGC0000149 | Spirangien\_biosynthetic\_gene\_cluster | 30 | 31.7675159236 | 268 | 7.2e-71 |
| AKQ22698.1 | malonyl\_CoA-acyl\_carrier\_protein\_transacylase | BGC0001186 | Misakinolide\_biosynthetic\_gene\_cluster | 32 | 24.2834394904 | 268 | 9.4e-71 |
| ADN68476.1 | SorA | BGC0000184 | Sorangicin\_biosynthetic\_gene\_cluster | 33 | 23.7261146497 | 268 | 9.4e-71 |
| AIJ04681.1 | polyketide\_synthase | BGC0001383 | Macrolactin\_biosynthetic\_gene\_cluster | 33 | 22.6114649682 | 268 | 9.4e-71 |
| ABC36687.1 | polyketide\_synthase | BGC0000964 | Burkholdac\_biosynthetic\_gene\_cluster | 35 | 20.6608280255 | 267 | 1.2e-70 |
| AJW65409.1 | type\_I\_modular\_polyketide\_synthase | BGC0001195 | Nocardiopsin\_biosynthetic\_gene\_cluster | 33 | 28.1847133758 | 267 | 1.2e-70 |
| CUW01173.1 | Type\_I\_polyketide\_synthase | BGC0001359 | PM100117\_/\_PM100118\_biosynthetic\_gene\_cluster | 30 | 32.6433121019 | 267 | 1.2e-70 |
| ABC84465.1 | NigAX | BGC0000114 | Nigericin\_biosynthetic\_gene\_cluster | 32 | 26.7914012739 | 266 | 3.6e-70 |
| CAL69893.1 | RhiE\_protein | BGC0001112 | Rhizoxins\_biosynthetic\_gene\_cluster | 34 | 21.6162420382 | 265 | 4.7e-70 |
| ABB86408.1 | GelA | BGC0000067 | Geldanamycin\_biosynthetic\_gene\_cluster | 30 | 32.1257961783 | 265 | 6.1e-70 |
| ACY01401.1 | AT-less\_polyketide\_synthase | BGC0000083 | Lactimidomycin\_biosynthetic\_gene\_cluster | 34 | 21.6560509554 | 265 | 8e-70 |
| ACY01400.1 | AT-less\_polyketide\_synthase | BGC0000083 | Lactimidomycin\_biosynthetic\_gene\_cluster | 33 | 22.173566879 | 264 | 1.4e-69 |
| CAL69890.1 | RhiC\_protein | BGC0001112 | Rhizoxins\_biosynthetic\_gene\_cluster | 34 | 22.0541401274 | 264 | 1.4e-69 |
| AAS98784.1 | JamM | BGC0001001 | Jamaicamide\_biosynthetic\_gene\_cluster | 26 | 36.1863057325 | 264 | 1.8e-69 |
| IF55\_RS32375 | beta-ketoacyl\_synthase | BGC0001348 | JBIR-100\_biosynthetic\_gene\_cluster | 35 | 24.800955414 | 264 | 1.8e-69 |
| ABF89568.1 | polyketide\_synthase | BGC0001025 | Myxovirescin\_biosynthetic\_gene\_cluster | 33 | 21.9347133758 | 264 | 1.8e-69 |
| AHE80994.1 | PieA4 | BGC0001169 | Piericidin\_A1\_biosynthetic\_gene\_cluster | 29 | 33.4792993631 | 263 | 2.3e-69 |
| CAJ76298.1 | putative\_hybrid\_polyketide-non-ribosomal\_peptide\_synthetase | BGC0000972 | Colibactin\_biosynthetic\_gene\_cluster | 27 | 35.0318471338 | 263 | 2.3e-69 |
| AAY32965.1 | DszB | BGC0001093 | Disorazole\_biosynthetic\_gene\_cluster | 33 | 22.8503184713 | 263 | 2.3e-69 |
| AAS98783.1 | JamL | BGC0001001 | Jamaicamide\_biosynthetic\_gene\_cluster | 26 | 36.4649681529 | 263 | 2.3e-69 |
| AAO06916.1 | GdmAI | BGC0000066 | Geldanamycin\_biosynthetic\_gene\_cluster | 29 | 32.8821656051 | 262 | 6.8e-69 |
| ADH04682.1 | polyketide\_synthase | BGC0001344 | Tubulysin\_biosynthetic\_gene\_cluster | 28 | 38.5350318471 | 261 | 8.8e-69 |
| AAN85523.1 | polyketide\_synthase | BGC0001101 | Leinamycin\_biosynthetic\_gene\_cluster | 32 | 24.4426751592 | 260 | 2e-68 |
| AAP42873.1 | NanA11 | BGC0000105 | Nanchangmycin\_biosynthetic\_gene\_cluster | 29 | 32.8025477707 | 260 | 2e-68 |
| AAZ77694.1 | ChlA2 | BGC0000036 | Chlorothricin\_biosynthetic\_gene\_cluster | 33 | 25.1194267516 | 260 | 2e-68 |
| AEZ53946.1 | polyketide\_synthase | BGC0000144 | Salinomycin\_biosynthetic\_gene\_cluster | 32 | 29.1003184713 | 260 | 2.6e-68 |
| AEZ53952.1 | polyketide\_synthase | BGC0000144 | Salinomycin\_biosynthetic\_gene\_cluster | 32 | 28.7818471338 | 260 | 2.6e-68 |
| AKQ22696.1 | malonyl\_CoA-acyl\_carrier\_protein\_transacylase | BGC0001186 | Misakinolide\_biosynthetic\_gene\_cluster | 33 | 22.4522292994 | 259 | 3.4e-68 |
| CAJ57409.1 | polyketide\_synthase\_type\_I | BGC0000176 | Difficidin\_biosynthetic\_gene\_cluster | 32 | 21.9347133758 | 259 | 3.4e-68 |
| BAK64638.1 | polyketide\_synthase | BGC0000135 | Reveromycin\_biosynthetic\_gene\_cluster | 29 | 32.5636942675 | 259 | 3.4e-68 |
| EED18001.1 | polyketide\_synthase,\_putative | BGC0000154 | Stipitatic\_acid\_biosynthetic\_gene\_cluster | 27 | 38.5748407643 | 259 | 3.4e-68 |
| AAY89053.1 | polyketide\_synthase | BGC0001069 | Chivosazole\_biosynthetic\_gene\_cluster | 33 | 21.9347133758 | 259 | 4.4e-68 |
| ACR13997.1 | modular\_polyketide\_synthase,\_type\_I\_PKS | BGC0000185 | Tartrolon\_biosynthetic\_gene\_cluster | 32 | 22.7707006369 | 259 | 5.7e-68 |
| CAN93351.1 | polyketide\_synthase | BGC0000179 | Etnangien\_biosynthetic\_gene\_cluster | 32 | 22.2133757962 | 258 | 7.5e-68 |
| ctg1\_orf254 |  | BGC0001200 | Lorneic\_acid\_A\_biosynthetic\_gene\_cluster | 32 | 27.9856687898 | 258 | 7.5e-68 |
| ACB46471.1 | polyketide\_synthase | BGC0000082 | Kijanimicin\_biosynthetic\_gene\_cluster | 30 | 28.5828025478 | 258 | 9.8e-68 |
| AAY89050.1 | polyketide\_synthase | BGC0001069 | Chivosazole\_biosynthetic\_gene\_cluster | 34 | 21.974522293 | 258 | 9.8e-68 |
| ABC34675.1 | polyketide\_synthase,\_putative | BGC0000186 | Thailandamide\_biosynthetic\_gene\_cluster | 33 | 21.9347133758 | 257 | 1.3e-67 |
| CAQ52622.1 | type\_I\_polyketide\_synthase,\_modules\_4-5 | BGC0001066 | Kendomycin\_biosynthetic\_gene\_cluster | 29 | 34.1958598726 | 257 | 1.3e-67 |
| ALA62323.1 | methylphloroacetophenone\_synthase | BGC0001309 | Usnic\_acid\_biosynthetic\_gene\_cluster | 28 | 32.0063694268 | 257 | 1.7e-67 |
| ABW96541.1 | type\_I\_modular\_polyketide\_synthase | BGC0000159 | Tautomycin\_biosynthetic\_gene\_cluster | 31 | 27.9458598726 | 255 | 4.8e-67 |
| ABI91465.1 | beta-ketoacyl\_synthase | BGC0001094 | Enacyloxin\_biosynthetic\_gene\_cluster | 29 | 28.025477707 | 255 | 6.3e-67 |
| AEH42473.1 | polyketide\_synthase | BGC0000032 | Calcimycin\_biosynthetic\_gene\_cluster | 31 | 26.9506369427 | 255 | 6.3e-67 |
| AFX60332.1 | polyketide\_synthase | BGC0001032 | Oocydin\_A\_biosynthetic\_gene\_cluster | 32 | 21.5366242038 | 255 | 6.3e-67 |
| CAG23977.1 | polyketide\_synthase\_type\_I | BGC0000176 | Difficidin\_biosynthetic\_gene\_cluster | 31 | 23.0891719745 | 255 | 8.3e-67 |
| AGS06823.1 | type\_I\_polyketide\_synthase,\_PedI-like\_protein | BGC0001092 | Diaphorin\_biosynthetic\_gene\_cluster | 33 | 22.1337579618 | 255 | 8.3e-67 |
| ABM63527.1 | BryB | BGC0000174 | Bryostatin\_biosynthetic\_gene\_cluster | 31 | 24.5621019108 | 254 | 1.1e-66 |
| CAQ64690.1 | lasalocid\_modular\_polyketide\_synthase | BGC0000087 | Lasalocid\_biosynthetic\_gene\_cluster | 30 | 28.1847133758 | 254 | 1.1e-66 |
| BAG85030.1 | putative\_polyketide\_synthase | BGC0000086 | Lasalocid\_biosynthetic\_gene\_cluster | 30 | 28.1847133758 | 254 | 1.1e-66 |
| BAR73007.1 | putative\_PKS\_(ACP-KS-AT-DH-KR-ACP-KS-AT-DH-ER-KR-ACP) | BGC0001194 | Hitachimycin\_biosynthetic\_gene\_cluster | 29 | 33.2404458599 | 254 | 1.4e-66 |
| AIU36103.1 | LglD | BGC0000180 | Legioliulin\_biosynthetic\_gene\_cluster | 34 | 21.2977707006 | 253 | 1.8e-66 |
| ctg1\_orf23 |  | BGC0001013 | Meridamycin\_biosynthetic\_gene\_cluster | 29 | 32.8821656051 | 253 | 1.8e-66 |
| ABC87512.1 | polyketide\_synthase | BGC0001011 | Meridamycin\_biosynthetic\_gene\_cluster | 29 | 32.8821656051 | 253 | 1.8e-66 |
| ctg1\_orf253 |  | BGC0001200 | Lorneic\_acid\_A\_biosynthetic\_gene\_cluster | 35 | 21.2977707006 | 253 | 1.8e-66 |
| CAQ64687.1 | lasalocid\_modular\_polyketide\_synthase | BGC0000087 | Lasalocid\_biosynthetic\_gene\_cluster | 29 | 34.3550955414 | 253 | 2.4e-66 |
| BAG85027.1 | putative\_polyketide\_synthase | BGC0000086 | Lasalocid\_biosynthetic\_gene\_cluster | 29 | 34.3550955414 | 253 | 2.4e-66 |
| ABM63528.1 | BryC | BGC0000174 | Bryostatin\_biosynthetic\_gene\_cluster | 33 | 21.6162420382 | 253 | 2.4e-66 |
| AFV30250.1 | polyketide\_synthase | BGC0000075 | Hygrocin\_biosynthetic\_gene\_cluster | 27 | 34.3949044586 | 253 | 2.4e-66 |
| AAY28225.1 | HbmAI | BGC0000074 | Herbimycin\_biosynthetic\_gene\_cluster | 29 | 32.8821656051 | 253 | 3.1e-66 |
| AFX60313.1 | polyketide\_synthase | BGC0001031 | Oocydin\_A\_biosynthetic\_gene\_cluster | 32 | 22.4522292994 | 252 | 4.1e-66 |
| CAQ52623.1 | type\_I\_polyketide\_synthase,\_module\_6 | BGC0001066 | Kendomycin\_biosynthetic\_gene\_cluster | 32 | 27.3487261146 | 252 | 7e-66 |
| CAJ57411.1 | polyketide\_synthase\_type\_I | BGC0000176 | Difficidin\_biosynthetic\_gene\_cluster | 33 | 21.8949044586 | 250 | 2.7e-65 |
| ABB90282.1 | polyketide\_synthase | BGC0001057 | Zearalenone\_biosynthetic\_gene\_cluster | 34 | 20.8996815287 | 248 | 7.7e-65 |
| ACD39753.1 | non-reducing\_polyketide\_synthase | BGC0000076 | Hypothemycin\_biosynthetic\_gene\_cluster | 31 | 22.3328025478 | 248 | 7.7e-65 |
| ACD39762.1 | non-reducing\_polyketide\_synthase | BGC0000077 | Hypothemycin\_biosynthetic\_gene\_cluster | 31 | 22.3328025478 | 248 | 7.7e-65 |
| CBJ89764.1 | Polyketide\_synthase\_involved\_in\_xenocoumacin\_synthesis | BGC0001054 | Xenocoumacin\_biosynthetic\_gene\_cluster | 26 | 38.9729299363 | 248 | 1e-64 |
| CAN93352.1 | polyketide\_synthase | BGC0000179 | Etnangien\_biosynthetic\_gene\_cluster | 30 | 24.9203821656 | 247 | 1.7e-64 |
| BAF50722.1 | polyketide\_synthase | BGC0001116 | Virginiamycin\_biosynthetic\_gene\_cluster | 34 | 20.4219745223 | 244 | 1.1e-63 |
| AEE88279.1 | CurK | BGC0000976 | Curacin\_biosynthetic\_gene\_cluster | 28 | 29.1799363057 | 244 | 1.5e-63 |
| AAT70106.1 | CurK | BGC0001165 | Curacin\_biosynthetic\_gene\_cluster | 28 | 29.1799363057 | 244 | 1.5e-63 |
| WP\_010639241.1 | beta-ketoacyl\_synthase | BGC0000958 | Antimycin\_biosynthetic\_gene\_cluster | 27 | 38.0175159236 | 243 | 1.9e-63 |
| CCP20048.1 | divL1\_protein | BGC0001119 | Divergolide\_biosynthetic\_gene\_cluster | 28 | 32.6831210191 | 242 | 5.5e-63 |
| AGC09499.1 | LobS4 | BGC0001183 | Lobophorin\_biosynthetic\_gene\_cluster | 30 | 27.826433121 | 242 | 5.5e-63 |
| AJO72742.1 | Type\_I\_modular\_polyketide\_synthase | BGC0001381 | Brasilinolide\_biosynthetic\_gene\_cluster | 30 | 29.4585987261 | 242 | 7.2e-63 |
| ctg1\_orf29 |  | BGC0000096 | Midecamycin\_biosynthetic\_gene\_cluster | 29 | 33.1210191083 | 242 | 7.2e-63 |
| ABM63529.1 | BryX | BGC0000174 | Bryostatin\_biosynthetic\_gene\_cluster | 31 | 22.7308917197 | 240 | 2.1e-62 |
| CCA89327.1 | trans-AT\_type\_I\_polyketide\_synthase | BGC0001111 | Rhizopodins\_biosynthetic\_gene\_cluster | 33 | 21.5366242038 | 239 | 3.6e-62 |
| AAC69330.1 | type\_I\_polyketide\_synthase\_PikAII | BGC0000094 | Methymycin\_/\_pikromycin\_biosynthetic\_gene\_cluster | 28 | 32.1656050955 | 239 | 3.6e-62 |
| EAA65602.1 | hypothetical\_protein | BGC0000022 | Asperfuranone\_biosynthetic\_gene\_cluster | 27 | 39.2515923567 | 237 | 2.3e-61 |
| AGI99482.1 | Type\_I\_polyketide\_synthase | BGC0001004 | Lobophorin\_biosynthetic\_gene\_cluster | 35 | 20.1831210191 | 236 | 3e-61 |
| CAJ57410.1 | polyketide\_synthase\_type\_I | BGC0000176 | Difficidin\_biosynthetic\_gene\_cluster | 30 | 24.6417197452 | 235 | 8.9e-61 |
| CAE45671.1 | borrelidin\_polyketide\_synthase,\_type\_I | BGC0000031 | Borrelidin\_biosynthetic\_gene\_cluster | 30 | 28.9808917197 | 234 | 1.5e-60 |
| ABK32260.1 | AmbF | BGC0000014 | Ambruticin\_biosynthetic\_gene\_cluster | 30 | 24.2834394904 | 233 | 2e-60 |
| AGY62754.1 | EbeB | BGC0000051 | Ebelactone\_biosynthetic\_gene\_cluster | 28 | 32.9617834395 | 233 | 2.6e-60 |
| BAP05593.1 | CalE | BGC0000967 | Calyculin\_biosynthetic\_gene\_cluster | 32 | 21.576433121 | 233 | 3.4e-60 |
| CAJ87591.1 | putative\_peptide/polyketide\_synthetase | BGC0001055 | Yersiniabactin\_biosynthetic\_gene\_cluster | 29 | 31.0907643312 | 231 | 1.3e-59 |
| CAA73127.1 | HMWP1\_protein | BGC0000467 | Yersiniabactin\_biosynthetic\_gene\_cluster | 29 | 31.0907643312 | 230 | 2.2e-59 |
| ACY13415.1 | KR\_domain\_protein | BGC0001367 | Haliamide\_biosynthetic\_gene\_cluster | 30 | 31.4888535032 | 230 | 2.8e-59 |
| ACR33077.1 | polyketide\_synthase | BGC0000017 | Anatoxin\_biosynthetic\_gene\_cluster | 30 | 20.8200636943 | 228 | 1.1e-58 |
| AKA54627.1 | PKS | BGC0001216 | Splenocin\_biosynthetic\_gene\_cluster | 27 | 38.0175159236 | 227 | 1.8e-58 |
| AEH42491.1 | polyketide\_synthase | BGC0000032 | Calcimycin\_biosynthetic\_gene\_cluster | 28 | 32.8025477707 | 226 | 3.2e-58 |
| ABS90478.1 | PKS | BGC0001106 | Oxazolomycin\_biosynthetic\_gene\_cluster | 30 | 23.6863057325 | 225 | 7e-58 |
| AGZ15474.1 | putative\_type\_I\_polyketide\_synthase | BGC0001036 | Polyoxypeptin\_biosynthetic\_gene\_cluster | 30 | 28.025477707 | 225 | 9.2e-58 |
| ADH01663.1 | putative\_polyketide\_synthase\_PKS3 | BGC0000099 | Monascorubrin\_biosynthetic\_gene\_cluster | 28 | 22.6910828025 | 222 | 4.5e-57 |
| AAU04878.1 | polyketide\_synthase | BGC0000365 | Glycopeptidolipid\_biosynthetic\_gene\_cluster | 31 | 26.1942675159 | 219 | 3.9e-56 |
| ABC84457.1 | NigAII | BGC0000114 | Nigericin\_biosynthetic\_gene\_cluster | 28 | 32.4044585987 | 218 | 8.6e-56 |
| ACY06286.1 | polyketide\_synthase | BGC0001042 | Sanglifehrin\_A\_biosynthetic\_gene\_cluster | 29 | 33.0414012739 | 210 | 3.1e-53 |
| AEH42474.1 | polyketide\_synthase | BGC0000032 | Calcimycin\_biosynthetic\_gene\_cluster | 30 | 24.8805732484 | 208 | 8.9e-53 |
| ACG60781.1 | PKS(KS/AT/MT/KR/ACP) | BGC0001058 | Zorbamycin\_biosynthetic\_gene\_cluster | 30 | 26.9904458599 | 207 | 1.5e-52 |
| ACI12948.1 | PfaA | BGC0000861 | Eicosapentaenoic\_acid\_biosynthetic\_gene\_cluster | 25 | 37.2213375796 | 207 | 2e-52 |
| ACR50791.1 | putative\_polyketide\_synthase | BGC0000163 | Tetronasin\_biosynthetic\_gene\_cluster | 31 | 21.9347133758 | 201 | 1.4e-50 |
| ABF92824.1 | polyketide\_synthase | BGC0001025 | Myxovirescin\_biosynthetic\_gene\_cluster | 27 | 30.3343949045 | 200 | 2.4e-50 |
| ALP32043.1 | CycC | BGC0001293 | Cyclizidine\_biosynthetic\_gene\_cluster | 30 | 27.2691082803 | 200 | 3.2e-50 |
| BAF02924.1 | type\_I\_polyketide\_synthase | BGC0000073 | Halstoctacosanolide\_biosynthetic\_gene\_cluster | 30 | 28.622611465 | 193 | 2.3e-48 |
| AAK57185.1 | MxaB1 | BGC0001022 | Myxalamid\_biosynthetic\_gene\_cluster | 26 | 28.0652866242 | 192 | 5e-48 |
| CBK62718.1 |  | BGC0001115 | Thiomarinol\_biosynthetic\_gene\_cluster | 28 | 21.7356687898 | 186 | 2.8e-46 |
| AKD43753.1 | HerB | BGC0001349 | Heronamide\_biosynthetic\_gene\_cluster | 28 | 24.1640127389 | 142 | 7.8e-33 |
| ACO94460.1 | polyketide\_synthase\_type\_I | BGC0000029 | BE-14106\_biosynthetic\_gene\_cluster | 27 | 25.4777070064 | 141 | 1.3e-32 |
| AGC09485.1 | LobS2 | BGC0001183 | Lobophorin\_biosynthetic\_gene\_cluster | 25 | 29.1799363057 | 139 | 3.9e-32 |
| BAQ21946.1 | putative\_type\_I\_polyketide\_synthase | BGC0001204 | Versipelostatin\_biosynthetic\_gene\_cluster | 25 | 27.3487261146 | 136 | 4.3e-31 |
| ACO94499.1 | polyketide\_synthase\_type\_I | BGC0000097 | ML-449\_biosynthetic\_gene\_cluster | 27 | 24.3630573248 | 133 | 4.7e-30 |
| AHH99919.1 | PKS\_I | BGC0000002 | Aculeximycin\_biosynthetic\_gene\_cluster | 27 | 27.5079617834 | 132 | 8.1e-30 |
| AKA59448.1 | polyketide\_synthase | BGC0001203 | Clarexpoxcin\_biosynthetic\_gene\_cluster | 25 | 37.8184713376 | 131 | 1.1e-29 |
